# Modulation of thermal noise and spectral sensitivity in Lake Baikal cottoid fish rhodopsins

**DOI:** 10.1038/srep38425

**Published:** 2016-12-09

**Authors:** Hoi Ling Luk, Nihar Bhattacharyya, Fabio Montisci, James M. Morrow, Federico Melaccio, Akimori Wada, Mudi Sheves, Francesca Fanelli, Belinda S. W. Chang, Massimo Olivucci

**Affiliations:** 1 Chemistry Department, Bowling Green State University, Bowling Green OH 43403, USA; 2 Department of Ecology and Evolutionary Biology and Department of Cell and Systems Biology, University of Toronto, 25 Harbord St., Toronto, ON M5S 3G5, Canada; 3 Dipartimento di Biotecnologie, Chimica e Farmacia, Università di Siena, Siena, I-53100, Italy; 4 Department of Organic Chemistry for Life Science, Kobe Pharmaceutical University, 4-19-1 Motoyamakita-machi, Higashinada-ku, Kobe 658-8558, Japan; 5 Department of Organic Chemistry, Weizman Institute of Science, 234 Herzl Street, Rehovot 7610001, Israel; 6 Department of Life Sciences, University of Modena and Reggio Emilia, I-41125 Modena, Italy

## Abstract

Lake Baikal is the deepest and one of the most ancient lakes in the world. Its unique ecology has resulted in the colonization of a diversity of depth habitats by a unique fauna that includes a group of teleost fish of the sub-order Cottoidei. This relatively recent radiation of cottoid fishes shows a gradual blue-shift in the wavelength of the absorption maximum of their visual pigments with increasing habitat depth. Here we combine homology modeling and quantum chemical calculations with experimental *in vitro* measurements of rhodopsins to investigate dim-light adaptation. The calculations, which were able to reproduce the trend of observed absorption maxima in both A1 and A2 rhodopsins, reveal a Barlow-type relationship between the absorption maxima and the thermal isomerization rate suggesting a link between the observed blue-shift and a thermal noise decrease. A Nakanishi point-charge analysis of the electrostatic effects of non-conserved and conserved amino acid residues surrounding the rhodopsin chromophore identified both close and distant sites affecting simultaneously spectral tuning and visual sensitivity. We propose that natural variation at these sites modulate both the thermal noise and spectral shifting in Baikal cottoid visual pigments resulting in adaptations that enable vision in deep water light environments.

Lake Baikal is located in Eastern Siberia and it is the deepest (1600 m) lake in the world. It holds approximately one fifth of the world’s liquid freshwater. A unique feature of the lake is that oxygenation levels in even the deepest regions do not fall below 75–80% of the surface levels^1^. This has enabled the colonization of all depth habitats by fauna that includes a flock of teleost fish of the sub-order *Cottoidei*.

The Baikal cottoid fishes are an ideal system to study visual pigment evolution as both the rod and cone pigments in these fish show a gradual blue-shift in the wavelength of the absorption maxima (λ_max_) in relation to their habitat depth. For instance, the λ_max_ of the rod pigment (called rhodopsin) shifts from 516 nm in the species that colonizes the surface to the 484 nm in the deepest species. These λ_max_ shifts reflect, exclusively, variations in the amino acids interacting with the chromophore (Fig. 1a and b) as all the Lake Baikal cottoid fish utilize the same A1 chromophore (Fig. 1c)^2^. This is, for instance, in contrast to many freshwater teleosts where λ_max_ red-shifts are due to the A2 chromophore^3^,^4^.

Previous studies of the Lake Baikal cottoid fish rhodopsins (from now on Baikal rhodopsins) suggest that the ancestral cottoid species that colonized the lake likely had a rhodopsin with a λ_max_ of around 505 nm, similar to the sub-littoral species^5^. Variation in λ_max_ values among present day Baikal fishes likely arose as a result of subsequent amino acid substitutions in rhodopsin, but their adaptive consequences and possible underlying mechanisms remain unclear. In deep sea fish, the observed 470–480 nm λ_max_ is thought to be an adaptation to the blue-shifted spectral maximum of the available downwelling light^6^,^7^,^8^. However, these theories do not consider other aspects of rhodopsin function such as photosensitivity, which may be more important in dimly lit deepwater environments such as those found in Lake Baikal and the deep sea.

An alternative explanation for the λ_max_ blue-shift observed in the deeper Baikal rhodopsins may be based on the existence of a Barlow-like correlation^9^,^10^. This is an inverse proportionality relationship between rhodopsin λ_max_ and chromophore thermal isomerization rate. By competing with the photoisomerization triggering the rhodopsin function, the thermal isomerization must contribute to the thermal noise^11^,^12^ decreasing visual acuity^13^. When assuming the validity of such an isomerization-noise link, the Barlow correlation implies that the λ_max_ blue-shift of the abyssal rhodopsins would reflect the need to reduce the noise in habitats with low light intensities^5^,^14^.

Here we investigate the thermal noise hypothesis through a combination of multiconfigurational quantum chemistry (MCQC) calculations and experimental studies on a set of Baikal rhodopsins. More specifically, we demonstrate the existence of a Barlow correlation between λ_max_, expressed in terms of the chromophore vertical excitation energy (ΔE), and isomerization rate, related to the chromophore activation energy (E_a_^T^) and derive an atomistic model of the ΔE and E_a_^T^ variation. The results show that the amino acid substitutions found in the sequences of the selected Baikal rhodopsin set, modulate ΔE and E_a_^T^ simultaneously in an interdependent parallel fashion suggesting that a reduction in thermal noise may have evolved in Lake Baikal fish pigments as a dim-light adaptation for increased photosensitivity.

## Results and Discussion

**Figure 1:**
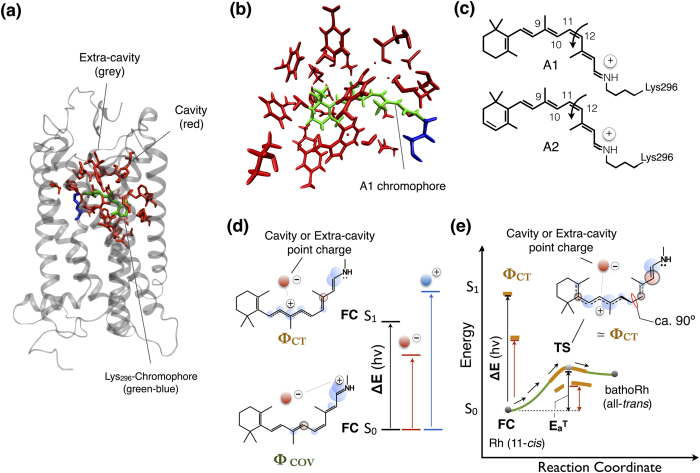
Rhodopsin structure and point-charge model. (**a**) Crystallographic structure of Rh. The amino acid of Rh belong either to the “cavity” or “extra-cavity” region. (**b**) Structure of the Rh Lys_296_-Chromophore (green-blue) and its cavity (red) comprising the conserved E113 counterion. (**c**) Structure of the A1 (11-*cis* retinal) and A2 (11-*cis* 3-dehydroretinal) chromophores. The arrows indicates the double-bond isomerization triggering the pigment function. (**d**) Effect of a negative (red) point charge located in proximity of the chromophore β-ionone ring. The charge would stabilize the electronically photo-excited state (S_1_) dominated by a charge-transfer electronic configuration (ϕ_CT_) with respect to the ground state (S_0_) dominated by a covalent electronic configuration (ϕ_COV_) leading to an increase in λ_max_ (i.e. a decrease in ΔE as shown in the red energy level diagram on the right). A charge of the opposite sign placed in the same location would lead to the opposite effect (blue energy level diagram). (**e**) Relationship between the ΔE (proportional to 1/λ_max_) and the E_a_^T^ controlling the chromophore thermal isomerization according to the point-charge model of ref. 8. A schematic representation of the chromophore charge distribution at the transition state (TS) is also given. The same negative/positive point charge would decrease/increase the barrier respectively.

The sequence similarity and marked λ_max_ variation of Baikal rhodopsins facilitate the study of the effect of single amino acid substitutions on ΔE and E_a_^T^. Accordingly, we consider four species representative of different habitats (in order of depth): the littoral (1–5 m) depth (*Paracottus kneri*, λ_max_ = 516 nm), the sub-littoral (1–120 m) depth (*Paracottus jettelesi*, λ_max_ = 505 nm), the supra-abyssal (50–450 m) depth (*Cottocomephorus inermis*, λ_max_ = 495 nm), and the abyssal (400–1500 m) depth (*Abyssocottus korotneffi*, λ_max_ = 484 nm)^5^. For each species a MCQC-based quantum-mechanics/molecular-mechanics (QM/MM) model of the corresponding rhodopsin is constructed (Methods section and *SI Appendix*, Fig. S1) using, as a template,the crystallographic structure of bovine rhodopsin (Rh). The model quality is assessed by reproducing: (i) the observed λ_max_ changes along the set plus the Rh template and (ii) the observed linear relationships between the λ_max_ of A1/A2 pairs of pigments^15^,^16^. This second test is carried out by preparing and spectroscopically characterizing *in vitro* Baikal rhodopsins where the A1 chromophore is replaced by the A2 chromophore forming red-shifted analogs^17^. The validated QM/MM models are then used to study the effects of the amino acid substitutions differentiating the four species through a computational implementation^18^ of the point-charge model proposed by Nakanishi and coworkers^19^ (Fig. 1d and e).

### Origin of the excitation energy changes

Three Lake Baikal pigments were expressed and purified *in vitro* with both the A1 and A2 chromophores. The measured λ_max_ of the A1 rhodopsins were found to be almost identical to the literature values measured via microspectroscopy (MSP)^2^. *C. inermis* λ_max_ was identical to MSP measurements (495 nm), while *A. korotneffi* was found to absorb maximally at 482 nm (−2 nm from MSP values) and *P. jettelesi* absorbed at 501 nm (−4 nm from MSP values) (*SI Appendix*, Fig. S2). As expected, the λ_max_ of the A2 rhodopsins was found to be red-shifted in comparison to the corresponding A1 rhodopsin value. *A. korotneffi* A2 pigment shifted to 499 nm, a total red-shift of 17 nm. *P. jettelesi* rhodopsin expressed with A2 chromophore shifted by 19 nm to 520 nm. C*. inermis* was red-shifted by 21 nm to 516 nm in the A2 pigment (*SI Appendix*, Fig. S2). All A2 rhodopsins were also successfully light bleached and their MII intermediate also showed the characteristic observed blue-shifted λ_max_ with respect to the dark adapted state (*SI Appendix*, Fig. S3) as expected for functional pigments.

**Figure 2:**
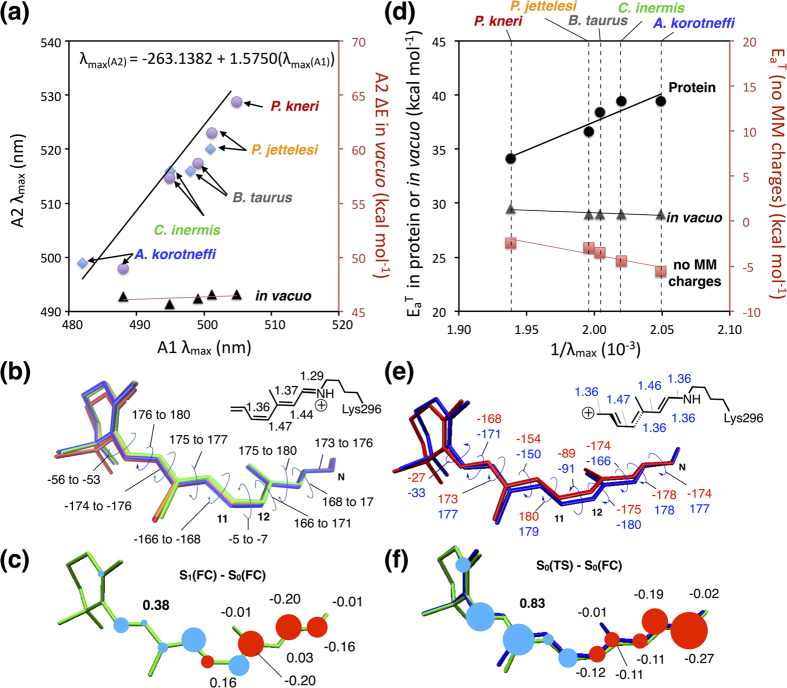
λ_max_ and E_a_^T^ values of Baikal cottoid fish rhodopsins. (**a**) Experimental (blue diamonds) and computed (purple circles) λ_max_ of the selected rhodopsins (the computed values are scaled by applying a factor of 1.03 and 1.05 to the corresponding ΔE of the A1 and A2 models respectively). The straight line indicates the linear relationship of 18 identical-opsin pairs selected by Dartnall and Lythgoe. The ΔE values for the isolated chromophores of the computed set are also displayed (black triangles). (**b**) Superimposed S_0_ equilibrium geometries of A1 retinal chromophores in *C. inermis* (green), *P. jettelesi* (orange), *A. korotneffi* (blue) and *P. kneri* (red). The relevant bond lengths and backbone dihedral angle are given in Å and degrees respectively. (**c**) Balloon diagram displaying the difference between the S_1_ and S_0_ charge (S_1_/S_0_ charge difference, in electron units) distributions at the S_0_ equilibrium structure of P. jettelesi. (**d**) Computed thermal isomerization E_a_^T^ (with a scaling factor of 1.18 applying to the A1 models, see SI for details) plotted as a function of the inverse of the corresponding λ_max_ for pigments with the A1 chromophores in the protein (black circles), isolated (gray triangles) and in the protein with no point charges (red squares). Straight lines indicate the ideal linear relationship. The significant difference between the computed 35–40 kcal mol^−1^ E_a_^T^ value (this work) and the ca. 22 kcal mol^−1^ value measured in the 288–298 K range assuming an Arrhenius kinetics has been discussed in ref. An updated discussion is given in the *SI Appendix* Section 6. (**e**) Transitions state geometries of the A1 retinal chromophore in *A. korotneffi* (blue) and *P. kneri* (red) rhodopsins. The relevant bond lengths and backbone dihedral angle are given in Å and degrees respectively. (**f**) Difference between the charge distributions between the S_0_ transition state structure and equilibrium structure for *P. jettelesi* (TS/S_0_ charge difference).

As reported in Fig. 2a (see also *SI Appendix*, Table S1), the observed A1 and A2 rhodopsin λ_max_ trends as well as the related A1/A2 linear relationship are reproduced by the QM/MM models. Furthermore, the computed A1/A2 slope only modestly deviate from that established experimentally by Dartnall and Lythgoe^15^ showing a 5 nm error (i.e. <1 kcal mol^−1^).

In order to investigate the origin of the λ_max_ trend, we computed the ΔE values (Fig. 2a and *SI Appendix*, Table S1) for the isolated (*in vacuo*) chromophores of the four Baikal pigments. In these computations, the geometrical parameters of the chromophore are fixed at the values of the S_0_ equilibrium structure of the QM/MM model. The results provide information on the ΔE variations due to the changes in chromophore geometry. Within the A1 and A2 sets, the ΔE values show only limited ≤1 kcal mol^−1^ variations consistently with the limited geometrical changes displayed in Fig. 2b (i.e. with dihedral angle changes ≤4 degrees). Thus, the model indicates that the λ_max_ variations are not due to progressive chromophore distortion (except for a fraction in the case of *C. inermis*) and must be dominated by electrostatic effects (i.e. by the variations in the point charges of cavity and extra-cavity amino acids).

### Effect of cavity and extra-cavity amino acids

The ΔE change between the most red-shifted model (*P. kneri*) and the most blue-shifted model (*A. korotneffi*) is 1.9 and 3.3 kcal mol^−1^ for the A1 and A2 chromophore respectively. This value (see Fig. 1d) reflects the stabilizing effect of the *P. kneri* and *A. korotneffi* protein environments on the difference in S_1_ and S_0_ charge distribution of the chromophore (the S_1_/S_0_ charge difference of Fig. 2c). Since the S_1_/S_0_ charge difference is similar in all pigments, we focused on the larger ΔE changes of the A2 rhodopsins.

**Figure 3:**
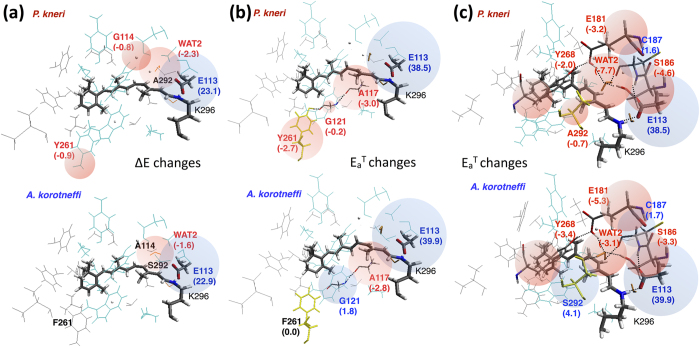
Effects of point charges of specific cavity residues between the two extreme cases: *P. kneri* and *A. korotneffi*. Apolar and polar residues are reported in gray and cyan, respectively and Gly residue (hydrogen) is shown as small gray sphere. The chromophore and the E113 counterion are shown in tube representation. The labels indicate residues that are not conserved in at least one of the four pigments of the cottoid fish set. (**a**) Retinal-binding pockets of the pigments with the A2 chromophore. ΔE-ΔE_off_ >0.5 kcal mol^−1^ in absolute value are labelled in red (negative shift) and blue (positive shift). The corresponding values are given in parenthesis in kcal mol^−1^ and represented by balloons. (**b**) Retinal-binding pockets of transition state with the A1 chromophores viewed with substitution (reported in yellow) at residue 261. E_a_^T^-E_a_^T^_off_ >0.5 kcal mol^−1^ in absolute value are labelled in red (negative shift) and blue (positive shift) and given in parenthesis in kcal mol^−1^. The dashed lines indicate hydrogen bonds. (**c**) The same data for the substitution of residue 292.

The ΔE decrease (red-shift) or increase (blue-shift) associated with a specific side-chain, can be evaluated by setting its point charges to zero and recomputing the excitation energy (ΔE_off_). The largest ΔE-ΔE_off_ differences computed for the cavity residues are displayed in the balloon diagrams of Fig. 3a. When comparing the effects of side-chain substitutions, one finds that a ΔE change may have two components. The first is a direct component due to the change in number, magnitude and position of the corresponding side-chain point charges. The second component is indirect and originates from the reorganization of the hydrogen bond network (HBN) induced by the same substitution. This second component/effect explains why conserved residues and water molecules may display large ΔE-ΔE_off_ changes and contribute to the total ΔE variation significantly.

When comparing the extreme cases of *P. kneri* (reddest) and *A. korotneffi* (bluest), the sequence data shows that the amino acid substitutions G114A and Y261F remove two red-shifting residues in *P. kneri* (see Fig. 3a) which directly contribute to blue-shifting the *A. korotneffi* absorption. While the same data shows that A292S does not change the ΔE-ΔE_off_, below we will see that this substitution modifies the HBN which then blue-shifts the λ_max_ indirectly. Thus variations in the composition of the rhodopsin cavity modulates the λ_max_ between littoral and abyssal habitats through direct and indirect changes. The same analysis indicates that, due to a cancellation of ΔE-ΔE_off_ of opposite signs (e.g. the sizable R140C red-shifting replacement is counterbalanced by the smaller T209I, L176S, T297S blue-shifting replacements in Fig. S6), the substitution of extra-cavity residues contributes only modestly to the λ_max_ change from *P. kneri* to *A. korotneffi*.

The sub-littoral and supra-abyssal species *P. jettelesi* and *C. inermis* feature the same amino acid cavity composition and similar cavity ΔE-ΔE_off_ values (*SI Appendix*, Fig. S6). In contrast, the extra-cavity substitutions T297S, D83N, T166S relating these species are associated with direct blue-shifting changes. This suggests that spectral tuning among species in the closer sub-littoral and supra-abyssal habitats may be controlled by extra-cavity amino acids. On the other hand, the ΔE variations computed between sub-littoral and littoral and between abyssal and supra-abyssal are modulated by both cavity and extra-cavity substitutions and by direct and indirect changes (*SI Appendix*, Table S4 and S5) as we will discuss below.

### Activation energy changes

In order to find out if the blue-shift observed when passing from the littoral to the abyssal habitat reflects the need to reduce the rhodopsin thermal noise, we built the QM/MM models for the S_0_ transition states (TS, Fig. 1e) that control thermal isomerization. The models allow to compute the corresponding E_a_^T^, thermal activation energy. The results yield a linear relationship between E_a_^T^ and 1/λ_max_ (Fig. 2d) with the most blue-shifted A1 rhodopsin (from *A. korotneffi*) displaying an E_a_^T^ 5.4 kcal mol^−1^ higher than the E_a_^T^ of the most red-shifted rhodopsin (from *P. kneri*). Notice that the present work is not aimed at reproducing the absolute values of the observed barriers but only their variation among different Baikal species. This is discussed in Section 6 of the SI Appendix which highlights a non-Arrhenius behavior as a source of discrepancy between computed and available observed E_a_^T^ values. In the same section, an additional source of inaccuracy is associated with the fact that reactant and transition state structures are computed as single points on the rhodopsin potential energy surface without explicitly accounting for the protein dynamics at body temperature. However, this error is expected to be systematic and therefore unable to affect the computed trends.

The geometrical structures of the chromophore at the TSs of *A. korotneffi* and *P. kneri* (see Fig. 2e) at the transition state are similar and consistent with those reported for Rh^18^. The structures indicate that the isomerization occurs via an aborted bicycle-pedal reaction coordinate^20^,^21^ involving the -C9=C10-C11=C12- segment of the chromophore backbone. Such motion is coupled with a substantially complete charge translocation from the =C12-C13=C14-C15=NH- segment to the segment containing the β-ionone ring (compare the schematic S_0_ reactant - i.e. the dark adapted state - and TS structures in Fig. 1d and e respectively).

Similar to what was found for ΔE, the E_a_^T^ of the chromophores *in vacuo,* i.e. the energy difference between the chromophores extracted from the QM/MM models of the TS and S_0_ reactant, are close (see Fig. 2d and Table S6). It is therefore concluded that the changes in E_a_^T^ are due to variations in the protein environment. Furthermore in Fig. 2d we show that electrostatic interactions prevail over steric (e.g. van der Waals) interactions. In order to isolate the steric effects, we zeroed all protein charges of the models and recomputed the E_a_^T^ values. The *A. korotneffi* value is found to be lower than the corresponding *P. kneri* value showing that steric effects would, as confirmed by the *P. jettelesi* and *C. inermis* E_a_^T^ values, result in a trend opposite to the one observed when both steric and electrostatic effects are considered (see also *SI Appendix*). It is thus concluded that the protein electrostatics determines the E_a_^T^ trend.

According to the point charge model, E_a_^T^ is modulated by the residue charges which “stabilize” or “destabilize” the TS/S_0_ charge changes (Fig. 2f). Such difference is qualitatively similar to the S_1_/S_0_ charge change (compare Fig. 2c and f). Thus, we investigate the differences in E_a_^T^ between *P. kneri* and *A. korotneffi* by applying the same analysis employed for ΔE. Accordingly, the effect of each residue is evaluated by computing the quantity E_a_^T^-E_a_^T^_off_ (E_a_^T^_off_ being the barrier obtained after zeroing the charges of a specific residue). When a residue is replaced such quantity is expected to display variations similar to the one seen for ΔE-ΔE_off_ but the significance of which is more complex to interpret. In fact, while ΔE-ΔE_off_ reflects, by definition, the effect of the residue charges, E_a_^T^-E_a_^T^_off_ also incorporates the effect of the geometrical difference between the TS and the S_0_ reactant. The E_a_^T^-E_a_^T^_off_ variations induced by the rhodopsin cavity substitutions relating *P. kneri* to *A. korotneffi* (Y261F, A292S and G114A), are given in Fig. 3b and c and Table S7. Y261F leads, through a direct change, to an increase of E_a_^T^ in *A. korotneffi* (2.7 kcal mol^−1^) consistently with the effect reported above for ΔE. As shown in Fig. 3c A292S leads, again through a direct change, to a large increase (4.8 kcal mol^−1^) in E_a_^T^ of *A. korotneffi*. Although this variation parallels the corresponding ΔE increase, the modeled E_a_^T^ change is due to HBN modification rather than a direct change as for ΔE. Finally, while G114A (see Fig. S7) leads to a negligible E_a_^T^ variation in *A. korotneffi*, it causes a limited direct ΔE increase (0.8 kcal mol^−1^). In conclusion, while the overall variation induced by the three substitutions show the same trend for both ΔE and E_a_^T^, their contributions may be mechanistically distinct as we detail below.

### Mechanisms of thermal noise modulation and spectral tuning

**Figure 4:**
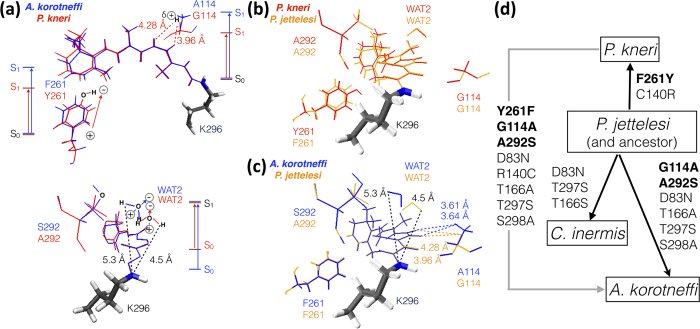
Spectral tuning sites in Baikal cottoid rhodopsins. (**a**) Direct (side-chain replacement) and indirect (conserved residue or water molecule reorientation) mechanisms for color-tuning based on Nakanishi point charge model. The effect of each mechanism on the ΔE variation (e.g. S_1_ destabilization or S_0_ stabilization) is illustrated by the corresponding bar diagrams. Top. Geometrical variations associated with the Y261F and G114A substitutions characterizing the transition from *P. kneri* (red) and *A. korotneffi* (blue). Bottom. Water molecule (WAT2) reorientation caused by the A292S substitution characterizing the same transition. (**b**) Spectral-tuning mechanism related to the Y261F substitution between *P. kneri* (red) and *P. jettelesi* (yellow). (**c**) Spectral-tuning mechanism related to the A292S and G114A substitutions between *P. jettelesi* and *A. korotneffi* (blue). (**d**) Cavity and extra-cavity substitutions associated with the transitions between different rhodopsins. The full arrows indicate the proposed evolutionary relationship between the corresponding species when assuming *P. jettelesi* to be the closest to the ancestor. In contrast, the grey arrow indicates the substitutions involved in the transition between littoral and abyssal species.

As reported above the combined effects of three cavity substitutions (see Fig. 4a) play a substantial role in establishing the differences between the ΔE and E_a_^T^ of *P. kneri* and *A. korotneffi*. The Y261F substitution blue-shifts the λ_max_ of all species relative to *P. kneri* by effectively changing the side-chain point charges. In fact, Y261F loses a dipole (the OH group of tyrosine) pointing its negative pole towards the β-ionone ring (see Fig. 4a top). This destabilizes the S_1_/S_0_ charge difference of Fig. 2c increasing the ΔE and leading to a blue-shift. As shown in Fig. 4b the same mechanism is seen when comparing *P. kneri* and *P. jettelesi*.

A parallel mechanism explains the increase of E_a_^T^ in *A. korotneffi*. with respect to *P. kneri*. In fact, similar to the ΔE effect, the Y261F substitution in *A. korotneffi* destabilizes the TS/S_0_ charge shift laid out in Fig. 2f and thus increases the E_a_^T^. However, in contrast to ΔE, E_a_^T^ is also modulated via an indirect effect of the same Y261F substitution. In fact, the loss of OH in position 261 in *A. korotneffi*, which used to form a hydrogen bond with the backbone oxygen of the conserved G121 residue in *P. kneri* (compare bottom and top in Fig. 3b), induces an HBN change. This change affects the stability of the TS and S_0_ reactant differently and contributes to the E_a_^T^ increase in *A. korotneffi*.

As seen in Fig. 3c, the A292S substitution relating *P. kneri* to *A. korotneffi* does not blue-shift the λ_max_ through a direct change, but through a modification of the HBN. In fact, A292S induces a relocation/reorientation of WAT2 which displaces it away from the Schiff base region (see Figs 3c and 4a bottom). Since the positive pole of WAT2 points towards the -C15=NH- moiety and destabilizes the S_1_/S_0_ charge difference, such WAT2 relocation increases the ΔE in *A. korotneffi*. The same mechanism, which is also responsible for the *P. jettelesi* to *A. korotneffi* λ_max_ blue-shift (see Fig. 4c), explains the increased E_a_^T^ in *A. korotneffi* through a decreased destabilization of the TS/S_0_ charge difference. However, the A292S induced WAT2 relocation also mediates a secondary indirect change of E_a_^T^. In fact, it perturbs an HBN connecting the conserved residues E181, S186 and Y268 (see bottom and top in Fig. 4b) which thus contribute to modulate E_a_^T^. This is demonstrated by the 1.3 kcal mol^−1^ increase of S186 and −2.1 and −1.4 kcal mol^−1^ decrease of E181 and Y268 respectively in *A. korotneffi* compared to *P. kneri*. Notice that, although individually E181 and Y268 induce a reduction in E_a_^T^, such HBN modulation is dominated by the 4.6 kcal mol^−1^ increase due to WAT2 (see Fig. 3c).

Finally, the G114A substitution, which replaces a non-polar residue with a sterically larger residue, shows a contrasting effect in *A. korotneffi*. As shown in Fig. 4a top and c, the G114 hydrogen of *P. kneri* and *P. jettelesi* is close to the Schiff base linkage and stabilizes the S_1_/S_0_ charge difference. Thus, the G114A substitution in *A. korotneffi* contributes to increase the ΔE. Such ΔE change is not paralleled E_a_^T^ which instead decreases. Nevertheless, due to the limited change in polarity, the decrease (see Fig. S7) is smaller than the E_a_^T^ increase due to the Y261F and A292S substitutions.

In conclusion, point-charge analysis has revealed a set of substitutions which simultaneously modulate ΔE and E_a_^T^ via cooperative direct and indirect HBN mediated mechanisms. While the magnitude of the described changes is expected to be sensitive to the details of our basic QM/MM models, the same substitutions have been detected in other contexts. In fact, Y261F has been shown to be responsible for the spectral differentiation between green and red cone pigments in primates^22^. G114A has been shown to cause a blue-shift also in Rh when expressed *in vivo*^23^,^24^ and in spite of the limited polarity change. A292S has also been detected in blue-shifted rhodopsin from other fish^25^, marine mammals^26^, and monotremes^27^.

### Light-sensitivity in related species

Above we have employed MCQC-based QM/MM models of rhodopsins reconstituted with both A1 and A2 retinals to investigate the relationship between spectral tuning and thermal isomerization rate in different species of cottoid fish. The results support the existence of a direct proportionality relationship between ΔE and E_a_^T^ for pigments of closely related species which evolved in the confined environment of Lake Baikal. This expands the validity of the Barlow correlation discussed for rod and cone pigments of distant species^9^,^10^,^28^ and provides a link with the observed inverse proportionality relationship between λ_max_ and isomerization rate in proton-pumping rhodopsins^29^ and even in the extreme case of 13-*cis* retinal chromophore salts in solution^30^.

The ΔE and E_a_^T^ proportionality originates at the electronic level. Indeed, the similarity between the S_1_/S_0_ and TS/S_0_ charge differences, (see Fig. 2c and f) due to the changes in chromophore π-electron density, makes ΔE and E_a_^T^ sensitive to the same substitutions. At a more fundamental level, such similarity originates from the fact that the same charge transfer configuration (ϕ_CT_) of the chromophore dominates the rhodopsin vertical S_1_ state and S_0_ transition state. As previously shown^18^, this is a consequence of a quantum mechanical property of the conical intersection of the rhodopsin chromophore^18^,^31^. Therefore the λ_max_ changes observed in Baikal rhodopsins reflects the biological exploitation of a quantum effect to increase light sensitivity^32^.

The analysis of the QM/MM models indicates that the variation of ΔE and E_a_^T^ in phylogenetically closely related rhodopsins is controlled by the electrostatic characteristics of the protein. Our implementation of Nakanishi’s point charge analysis has identified 8 rhodopsin substitutions, over a total of 20, modulating light sensitivity from red-shifted *P. kneri* to the blue-shifted *A. korotneffi*. The same analysis also produced an “atomistic model” of dim-light adaptation through specific side-chain substitutions. Through this model, specific mechanisms can be associated to the proposed phylogeny^5^ assumed to originate from *P. jettelesi* as its λ_max_ matches that of the ancestor. While the modification of the point charges associated with a cavity substitution have a direct impact on ΔE and E_a_^T^ (e.g. F261Y when comparing *P. jettelesi* and *P. kneri* in Fig. 4b), it would be impossible to model the observed trends without taking into account the HBN modifications associated with the same substitution (e.g. A292S comparing *P. jettelesi* and *P. korotneffi* in Fig. 4c) or the effect of extra-cavity substitutions (e.g. D83N, T297S and T166S when comparing *P. jettelesi* and *C. inermis* and, additionally, S298A in *P. jettelesi* and *A. korotneffi*). Also, in our QM/MM models, extra-cavity substitutions display large effects when an ionized residue replaces a neutral one (e.g. C140R replacing cysteine in *P. jettelesi* to a arginine in *P. kneri*).

In conclusion, when assuming that the thermal isomerization of rhodopsin dominates its thermal noise, the regular Baikal rhodopsin blue-shift observed when moving from littoral to abyssal habitats may be a byproduct of visual adaptations to extremely low levels of illumination. In fact, our study shows that for Baikal fishes, these two aspects of visual pigment function are interdependent: the isomerization rate (which would determine the amount of thermal noise) and the wavelength of maximal absorbance. Amino acid substitutions have evolved in these fishes that shift both quantities simultaneously for adaptations that would contribute to better visual sensitivity and enable colonization of the dimly lit blue-shifted deepwater environments of Lake Baikal. Our results suggest that it is possible similar mechanisms may underlie colonization of other deepwater dimly lit environments such as those inhabited by deep sea fishes in marine habitats.

## Methods

### Molecular biology methods

No experiments on live vertebrates were carried out in this study. Incomplete Baikal cottoid RH1 sequences were taken from^5^ and completed with wildtype bovine sequences for the N- and C-termini. The full-length hybrid RH1 genes were synthesized by GeneArt (Invitrogen) with *Bam*HI and *Eco*RI restriction sites at the 5′ and 3′ ends, respectively. The synthesized sequences were then inserted into the p1D4-hrGFP II expression vector which tags expressed rhodopsin sequences with the nine amino acid 1D4 peptide sequence (TETSQVAPA) at the carboxy terminus^33^. This enables immunoaffinity purification of expressed proteins from HEK293T cells as previously described^34^,^35^. UV-vis absorption spectra of purified rhodopsin samples were measured at room temperature both in the dark, and following light-bleaching for 60 seconds using a fiber optic lamp. Difference spectra were calculated by subtracting the light-bleached spectra from respective dark spectra. To provide accurate estimates of λ_max_, dark absorbance spectra were fit to standard templates for either A1 or A2 visual pigments^36^.

### Computational methods

The QM/MM models of both A1 and A2 fish rhodopsins were prepared starting with a structures obtained via comparative modeling. To do so, the chain A of the 1U19 structure of bovine rhodopsin^37^ was used as a template. The models were then constructed by relaxing the cavity-counterion-chromophore complex in its protein environment via molecular dynamics and geometry optimization. The chromophore was treated using the complete-active-space self-consistent field (CASSCF) method^38^ with an active space corresponding to the entire π-system and the 6–31G* basis set. The protein environment was instead described using the AMBER force field. To account for the dynamic electron correlation, the model equilibrium CASSCF/AMBER geometries and wavefunctions were used for single-point multiconfigurational second-order perturbation theory (CASPT2) calculations with a two-root state average zeroth-order wavefunction^39^. The ΔE values are computed at the CASPT2//CASSCF/AMBER level. The transition states controlling the thermal isomerization were located via restricted-step rational-function-optimizations^40^ at the CASSCF/AMBER level. The corresponding E_a_^T^ values were computed at the CASPT2//CASSCF/AMBER level. See the *SI Appendix* for further details.

## Additional Information

**How to cite this article**: Luk, H. L. *et al*. Modulation of thermal noise and spectral sensitivity in Lake Baikal cottoid fish rhodopsins. *Sci. Rep.* **6**, 38425; doi: 10.1038/srep38425 (2016).

**Publisher's note:** Springer Nature remains neutral with regard to jurisdictional claims in published maps and institutional affiliations.

## Supplementary Material

Supplementary Appendix
