# Supplementary material for: Modulation of thermal noise and spectral sensitivity in Lake Baikal cottoid fish rhodopsins
Source: Sci Rep. 2016 Dec 9;6:38425. doi: 10.1038/srep38425 (PMC5146971; doi:10.1038/srep38425)
Supplement: Supplementary Appendix [file srep38425-s1.pdf]

Supporting Appendix for:

Modulation of thermal noise and spectral sensitivity in Lake Baikal cottoid fish rhodopsins

Hoi Ling Luk[a], Nihar Bhattacharyya[b], Fabio Montisci[c], James M. Morrow[b], Federico Melaccio[c], Akimori Wada[d], Mudi Sheves[e], Francesca Fanelli[f], Belinda S. W. Chang[b], Massimo Olivucci[a,c]

[a]Chemistry Department, Bowling Green State University, Bowling Green OH 43403, USA
[b]Department of Ecology and Evolutionary Biology and Department of Cell and Systems Biology, University of Toronto, 25 Harbord St., Toronto, ON M5S 3G5, Canada.
[c]Dipartimento di Biotecnologie, Chimica e Farmacia, Università di Siena, Siena, I-53100, Italy
[d]Department of Organic Chemistry for Life Science, Kobe Pharmaceutical University, 4-19-1 Motoyamakita-machi, Higashinada-ku, Kobe 658-8558, Japan.
[e]Department of Organic Chemistry, Weizman Institute of Science, 234 Herzl Street, Rehovot 7610001, Israel
[f]Department of Life Sciences, University of Modena and Reggio Emilia, I-41125 Modena, Italy

1. QM/MM Models

1.1 Model constructions

Here we report on the detailed workflow for the construction of the selected (reference) quantum mechanics/molecular mechanics (QM/MM) models of Lake Baikal rhodopsins. Our target is to construct QM/MM models that can reproduce all the relevant observed spectroscopic data.

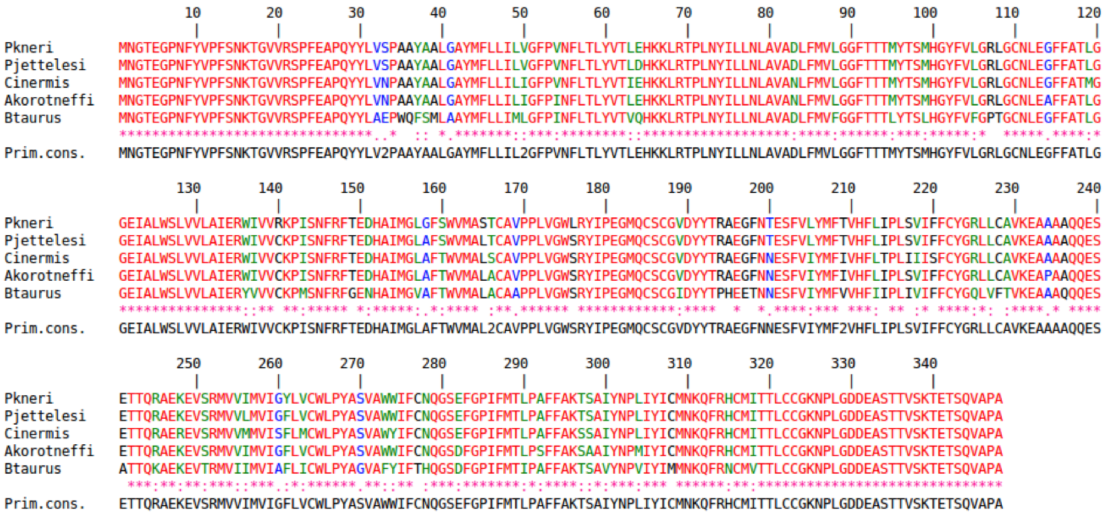

Figure S1. Sequence alignment of the selected lake Baikal cottoid fish rhodopsins and the template Rh (*B. taurus*). The alignment was performed with CLUSTALW.[1]

Given the lack of crystallographic structures, the eight QM/MM models of the selected fish rhodopsins were prepared on the basis of a comparative modelling approach. Accordingly, the crystallographic structure of bovine rhodopsin (*B. taurus*) (PDB code: 1U19, chain A),[2] was used both to construct the corresponding *B. taurus* QM/MM models with the A1 chromophores and as a template to generate the initial fish rhodopsin structures. Since the four fish rhodopsins display a sequence similarity greater than 80% when aligned to bovine rhodopsin, the comparative models were constructed by keeping fixed the protein backbone and then replacing the amino acid of the template with the corresponding amino acid as indicated in the aligned sequence. (See Fig. S1) The mutated side chains were subjected to rotamer adjustment by using the Dunbrack and Karplus[3], Ponder and Richards[4], and Sutcliffe rotamer libraries[5], implemented in the Quanta package.[6] Rotamer assignment privileged consensus values among the three different libraries, taking also into account the degree of fitting between mutated and original amino acid in the template structure. The structural models were then completed by adding 11-*cis* retinal and 21 water molecules, all extracted from the template crystallographic structure.

In order to have a neutral system overall, we set the total charge on the protein (MM subsystem) to -1 (the QM subsystem, which contains the chromophore bears a +1 charge due to protonated Schiff base linkage). Accordingly a number of chloride ions were added near positively charged residues (such as Arg, His) on the protein surface and far away from the chromophore. The positions of chloride ions were found to have a limited effect on the spectroscopic properties of the chromophore, as well as on test trajectories. Amino acid ionization states were determined with PROPKA 3.0.[7,8] In particular, we use a protonated form of E181 and E122. There are several conflicting experimental and computational conflicting studies on the protonation state of E181[9-14]. In our model, deprotonation of E181 leads to a ~60 nm blue-shift of the computed absorption maxima indicating an unbalanced/ incorrect electrostatic environment around the chromophore. This is consistent with other QM/MM reports where it was reported that deprotonation of the E181 leads to a large blue-shift (see the supporting information of[13] and also[12] and[14].

The retinal chromophore was treated quantum mechanically using the *ab initio* complete-active-space self consistent field (CASSCF) method. [15] The protein environment is described by the AMBER94 force field[16] with modified parameters for the Lys residue linked to the chromophore.[17,18] Electrostatic embedding was used to describe the interaction between the MM and QM subsystems using the ESPF concept.[19-21] CASSCF is a flexible multiconfigurational method for an unbiased description of the electronic character on both excited and ground state (i.e. with no empirical derived parameters and avoiding single-reference wavefunctions). Equilibrium and transition state structures were optimized at the single-root CASSCF(12,12)/6-31G*/AMBER level. QM/MM calculations were carried out with

Molcas 7.8[22] and Tinker 5.1[4] programs, within the microiterations approach.[21] The QM/MM boundary is at the Lys Cδ-Cε bond and the link-atom scheme was used to treat the frontier between the QM and MM subsystems.[23] The partitioning of QM and MM atoms was as follows:

- QM atoms: all retinal atoms, five atoms of the lysine chain connected to it (ε-nitrogen, ε-carbon and their hydrogen atoms and the link atom. There are 54 QM atoms in total.
- Explicit MM atoms: the remaining 9 atoms of the lysine side-chain are treated explicitly by Molcas (as opposed to other atoms which are treated by Tinker). These atoms are optimized along with the QM part and not by microiterations as the other MM atoms (see below).
- ACTIVE MM atoms: for ASR_{13C} and ASR_{AT} these are given by the union set of all the side-chains or waters that has at least one atom that is within 4 Å from the QM region. These MM atoms are optimized using the microiteration methods available in Molcas/Tinker.
- INACTIVE MM atoms: all the remaining atoms. These atoms contribute to the electrostatic environment around the chromophore but their geometries are kept frozen during QM/MM optimizations.

In order to generate the model hydrogen-bond network consistently, the hydrogen atoms were removed from the comparative models and successively added by DOWSER[24] for hydrogen atoms bound to crystallographic water. The remaining hydrogen atoms were added by Gromacs 4.5.5 and a molecular mechanics (MM) minimization was performed on these hydrogens. A list of the residues that constitutes the retinal chromophore binding pocket was obtained using "Computed Atlas of Surface Topography of proteins" (CASTp).[25] A probe radius of 1.4 Å was used for the calculation; the resulting retinal-binding pocket is composed of 27 amino acids. Molecular dynamics on amino acid side-chains were performed within the CASTp cavity. The MD run consists of a 50 ps heating followed by 150 ps equilibration and 800 ps production. The resulting model is used for subsequent QM/MM optimizations,[26,27] which were performed using Molcas/Tinker. It includes:

- A QM/MM Hartree-Fock/3-21G single point calculation was performed to calculate ESPF charges on QM atoms;
- Tinker minimization of ACTIVE MM atoms using the calculated ESPF charges, with the same settings used for hydrogen atoms minimization;
- QM/MM Molcas/Tinker HF/3-21G optimization of the system with ACTIVE atoms relaxed;
- QM/MM Molcas/Tinker CASSCF(12,12)/3-21G optimization of the system with ACTIVE atoms relaxed;
- QM/MM Molcas/Tinker CASSCF(12,12)/6-31G* optimization of the system with ACTIVE atoms relaxed.

The CASSCF geometries were used for subsequent multiconfigurational second-order perturbation

theory (CASPT2) single point energy computations[28] that allow for a more quantitative evaluation of the excitation energies and excited state energy differences by accounting for the dynamic electron correlation. The CASPT2 energies were computed with an imaginary shift of 0.2 to exclude possible intruder states, and with the IPEA shift[29] set to zero thus overriding the default value of 0.25. It has been shown that the CASPT2(IPEA=0)//CASSCF/6-31G* protocol (the double slash // indicates single-point calculations with the level used for geometry optimization indicated after it) yields excitation energies in better agreement with MRCISD+Q//CASPT2(IPEA=0.25)/ANO-L-VTZP reference computations than CASPT2(IPEA=0.25)//CASSCF/6-31G*, for a small model of the 11-*cis* retinal protonated Schiff base (PSB11) chromophore featuring three conjugated double bonds (PSB3).[30] This is due to a cancellation of errors which originates from opposite energy differences due to the geometries, correlation energies (i.e. method and IPEA correction) and basis sets with respect to the reference.[30] Since systematic CASPT2 minima and transition state geometry optimizations as well as trajectory computations (e.g. intercepting potential energy crossings) are currently unfeasible for a chromophore such as PSB11 (i.e. one is forced to use a CASSCF level), we used the CASPT2(IPEA=0)//CASSCF/6-31G*/AMBER protocol. This appears to be, presently, a viable compromise if equilibrium structures, excitation energies, trajectories, conical intersections and transition states with different electronic structures need to be computed on a common methodological basis and if the main focus is on mechanistic studies rather than quantitative studies. The final geometry optimization leads to a CASSCF(12,12)/6-31G*/AMBER equilibrium structure for all models.

The A2 chromophore models were constructed by using the modified A1 optimized models (by deleting the four hydrogens on C3 and C4 and put back two hydrogens such that C3 and C4 are planar.) as the starting geometry for the series of QM/MM optimizations using Molcas/Tinker and get the single point CASPT2 energies as described above except using the active space of (14,14).

Table S1 shows that the trend of the observed λ_{max} values is successfully reproduced using our QM/MM models after a correction factor of 1.03 for A1 models and 1.05 for A2 models is applied to all Baikal computed ΔE values. The need for these corrections is generically attributed to the systematic error generated during comparative modeling and QM/MM model construction. On the other hand, for both the A1 and A2 pigments, the computed ΔE changes are consistent with the experimental observations. And the observed linear correlation between the λ_{max} values of A1 and A2 pigments is computationally reproduced (see Fig. 2A and D in the main text) showing that the effect of the protein environment is similar for the two chromophores. Furthermore, the computed slope (trend) for this relationship seems only to modestly deviate from the the relationship established by Dartnall and Lythgoe[31] showing an error bar of 5 nm (<1 kcal mol^{-1}).

Table S1. Experimental and computed vertical excitation energies ΔE (kcal mol^{-1}) for the fish visual pigments and for the bovine rhodopsin (*B. taurus*) template used to generate the QM/MM models. The corresponding λ_{max} values (nm) are given in brackets. Errors are obtained by calculating the differences between the computed and observed values. The relative ΔE changes with respect to the pigment from *P. jettelesi* are reported in kcal mol^{-1}. All observed values are based on our experimental measurements (see Fig. S2 and S3 below) except the A1 measurement for *P. kneri* is based on ref. 33.

Species	A1 ΔE (λ_{max})				A2 ΔE (λ_{max})			
	Observed	Relative change	Computed	Relative change	Observed	Relative change	Computed	Relative change
P. kneri	55.4 (516)	-1.7	56.7 (505)	-0.4	N/A	N/A	54.1 (529)	-0.6
P. jettelesi	57.1 (501)	0.0	57.1 (501)	0.0	55.0 (520)	0.0	54.7 (523)	0.0
B. taurus	57.3 (499)	0.2	57.3 (499)	0.2	55.4 (516)	0.4	55.3 (517)	0.6
C. inermis	57.8 (495)	0.7	57.8 (495)	0.7	55.4 (516)	0.4	55.6 (515)	0.9
A. korotneffi	59.3 (482)	2.2	58.6 (488)	1.5	57.3 (499)	2.3	57.4 (498)	2.7

1.2 Transition state optimizations

The transition states were optimized using the restricted-step rational-function-optimization method at the CASSCF/6-31G*/AMBER level of theory.[32] Since QM/MM frequency analysis is unavailable in MOLCAS/Tinker, initial attempts to optimize a transition state had to rely on a guess Hessian computed at a suitable guess structure. The quality of the Hessian is evaluated by looking at the reaction vector, thus making sure that it describes the expected isomerization motion connecting the cis to trans structures. The optimizations were considered completed after convergence to a stationary point and the corresponding final updated transition vector represents the expected space-saving bicycle-pedal motion. All energy barriers are reported relative to the corresponding ground state optimized structure and are computed at the CASPT2//CASSCF/6-31G*/AMBER level of theory. In this case, CASPT2 with the default IPEA value of 0.25[29] was used since it has been shown to be more accurate for evaluating energy barriers,[30] (but not vertical excitation energies, as discussed above, since the factors leading to a cancellation of errors in the case of vertical excitation energies no longer apply at the transition states). For more details, we refer the reader to ref. 30 where the use of the CASPT2//CASSCF protocol was benchmarked for a reduced model of the retinal protonated Schiff base). For the three intermediate cases (*P. Jettelesi, B. Taurus and C. inermis*), the TS_{CT} could not be optimized because it is very close to the conical intersection. Instead, these transition state geometries are determined by inserting the chromophore of *A. korotneffi*, which is more closely resmebled than that of *P. kneri*, for these models with a MM miminization on the cavity.

Similar to the λ_{max} values, a correction factor of 1.18 is applied for all the A1 Baikal computed E_a^T. And again, the need for these corrections is generically attributed to the systematic error generated

during comparative modeling and QM/MM model construction. The different correction factors used can be accounted by the difference in the charge residing on the β-ionone moiety for the reactant excited state and TS_{CT}. (Table S2) These results show that there is a larger charge residing on β-ionone moiety for the TS_{CT} than in the reactant excited state. Hence, the corresponding vertical excitation energy would have a smaller correction factor (1.03 for A1 chromophore) than that used in the correcting the activation energy barrier (1.18)

Table S2. Total Mulliken charge resides on the β-ionone moiety for the reactant excited state and TS_{CT}. These values represent the degree of charge transfer for each model, and are computed by summing the charges on all the atoms which are on the β-ionone side of the isomerizing bond.

Charge on β-ionone moiety	reactant-S_1	TS_{CT}
P. kneri	0.54	1.00
P. jettelesi	0.53	0.98
B. taurus	0.51	0.97
C. inermis	0.49	0.97
A. korotneffi	0.48	0.97

2. Experimental results

Three lake Baikal pigments were successfully expressed and purified *in vitro* with both the A1 and A2 chromophores. The pigments from *A. korotneffi, C. inermis, and P. jettelesi* were all expressed with high enough yield for functional assays (see Fig. S2). When the λ_{max} values of the expressed A1 pigments were found to be almost identical to the literature values from MSP[33]. As expected, the λ_{max} of the A2 pigments were found to be redshifted in comparison to the corresponding A1 pigments. The pigments from *P. jettelesi* and *A. korotneffi* were both 15 nm redshifted by the A2 chromophore while *C. inermis* was 21 nm redshifted. All three pigments were also successfully light bleached (Fig. S3) and, again, their MII intermediate absorbance peak showed the characteristic red-shifted λ_{max} when A2 and A1 pigments were compared.

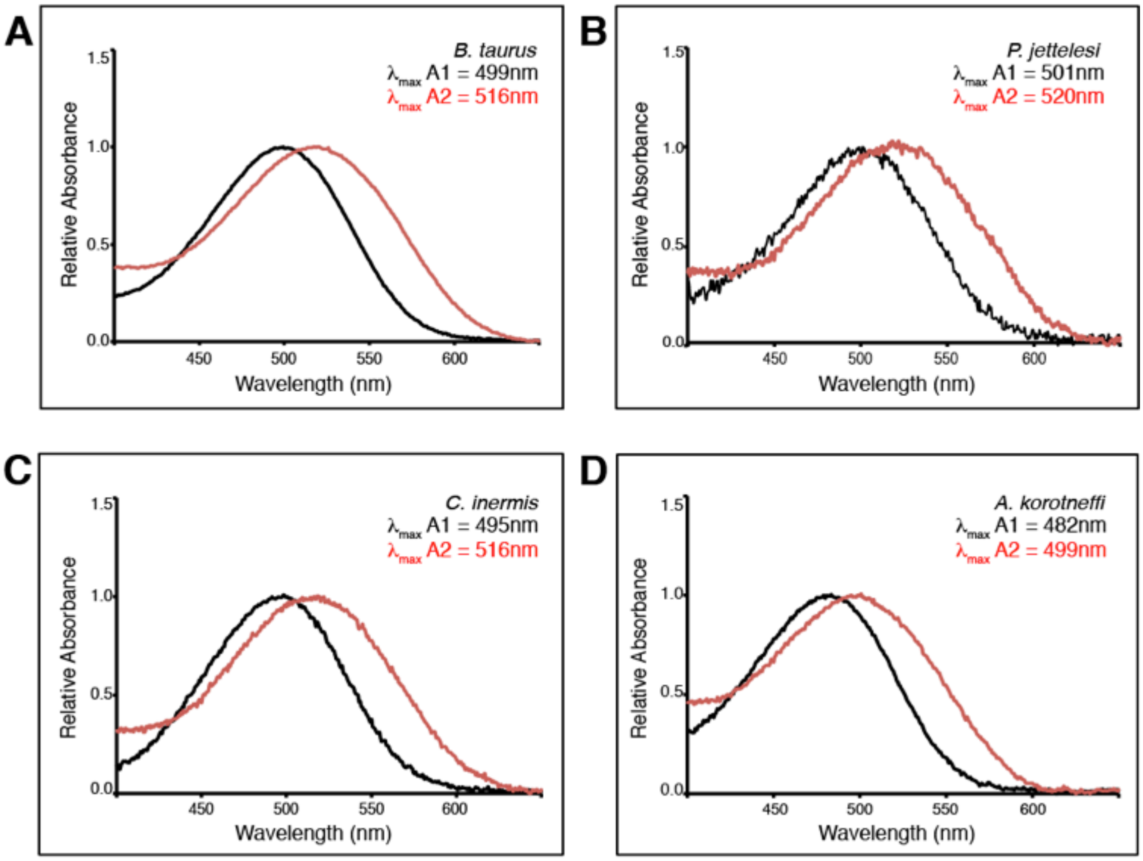

Figure S2. UV-vis absorbance spectra of in vitro expressed and purified Baikal cottoid and bovine Rh sequences regenerated with A1 and A2 chromophore. (A) wild type bovine (*B. taurus*) rhodopsin has a λ_{max} of 499 nm when regenerated with the A1 chromophore and 516 nm with the A2 chromophore. (B) *P. jettelesi* λ_{max} A1 is 501nm, similar to MSP values. λ_{max} A2 is redshifted to 520 nm. (C) *C. inermis* is slightly blue-shifted λ_{max} A1 at 495nm, but the λ_{max} A2 is 516nm. (D) *A. korotneffi* is the most blue-shifted A1 (482 nm) and A2 (499 nm) pigment.

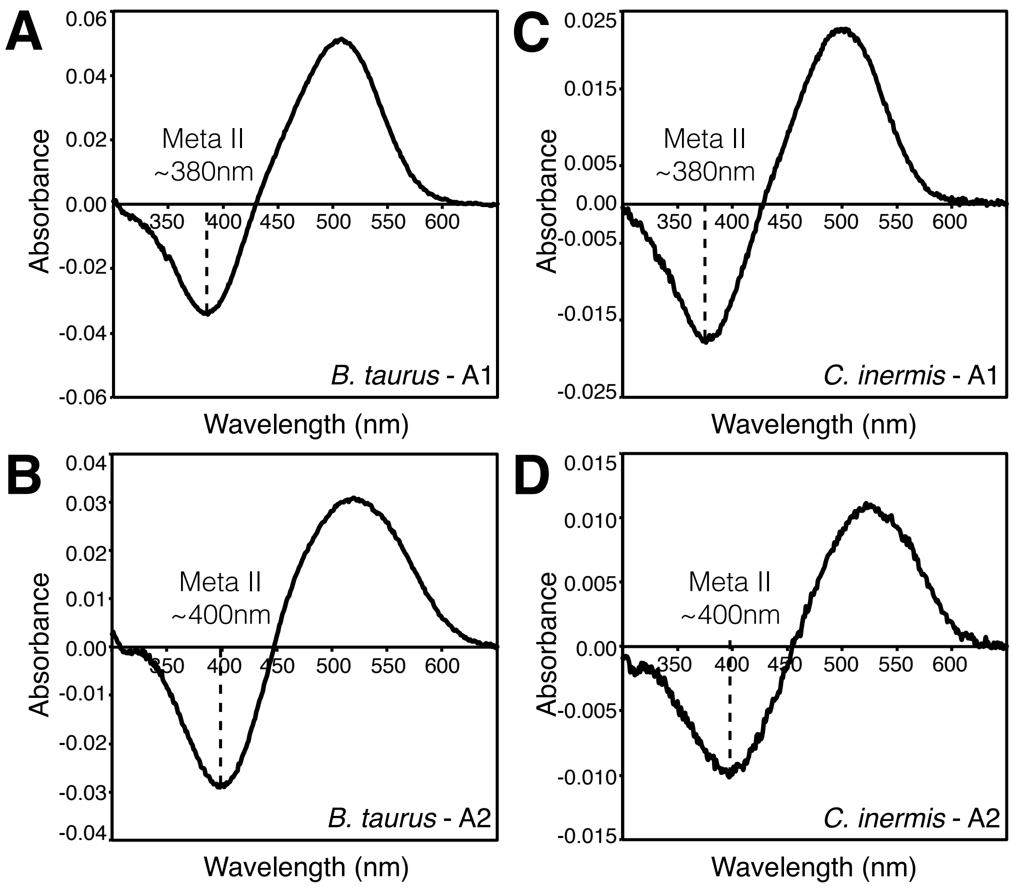

Figure S3. Dark-light difference spectra of wildtype bovine rhodopsin with (A) A1 chromophore and (B) A2 chromophore and C. inermis wildtype rhodopsin with (C) A1 chromophore and (D) A2 chromophore. Note that the Meta II absorbance of A2 pigments is also red-shifted from 380nm to ~400nm.

3. Electrostatic effects on spectral tuning.

In order to analyze the molecular-level factors controlling the ΔE changes computed with respect to *P. jettelesi*, these are decomposed according to three subsystems (I-III) (see Scheme S1). The QM subsystem I corresponding to the chromophore, the MM inner subsystem II and the MM outer subsystem III. In other words, we evaluate the contributions originating from the different geometry of the chromophore, as well as all CASTp cavity residues and all water molecules with at least one atom within 4 Å from any atom of the chromophore which constitutes the subsystem II.

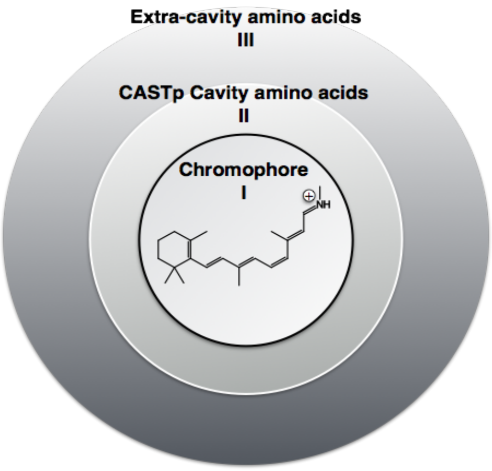

Scheme S1. Vertical excitation energy decomposition scheme. The three sub-systems are schematically illustrated by the chromophore structure and the two sets of cavity and extra-cavity amino acids.

The results reported in Table S3 show that the excitation energies for the A2 chromophore are shifted to the red with respect to those of the corresponding A1 chromophore due to the increase in the length of the conjugated chain. However, within the A1 and A2 sets the ΔE values show only tiny variations maybe with the only exception of *C. inermis* which displays a limited 1 kcal mol^{-1} red-shifted value with respect to the *P. jettelesi* reference pigment. These small differences are consistent with the limited geometrical variations displayed in Fig. S4 where the changes in the dihedral angles is, at most, 4 degrees. Thus it can be qualitatively concluded that, according to our models, the ΔE changes (and therefore λ_{max} variations) of the lake Baikal cottoid photoreceptors are not due to the chromophore geometry (except, partially, for the case of *C. inermis*) and must therefore be dominated by the electrostatics of the protein environment (i.e. of subsystems II and III in Scheme S1).

Table S3. Computed ΔE values in kcal mol^{-1} for the A1 and A2 chromophore *in vacuo* for the four selected fish rhodopsins and for the template bovine rhodopsin (*B. taurus*) relative to the reference from *P. jettelesi*. The absolute values are given in brackets.

chromophore	*P. kneri*	*P. jettelesi*	*B. taurus*	*C. inermis*	*A. korotneffi*
A1	-0.2	0.0	-0.2	-1.0	-0.2
	(51.2)	(51.4)	(51.2)	(50.4)	(51.2)
A2	-0.4	0.0	0.0	-0.9	-0.2
	(46.2)	(46.6)	(46.6)	(45.7)	(46.4)

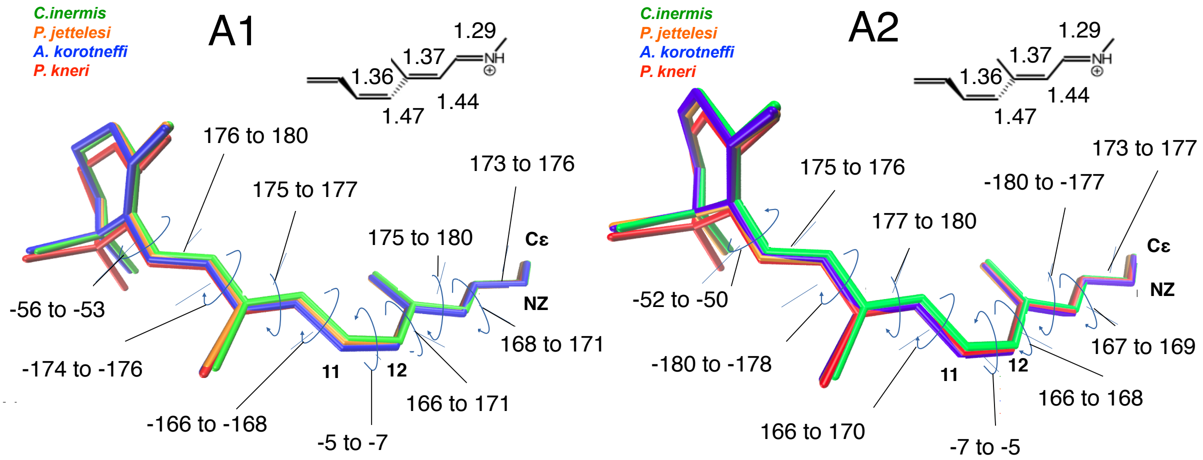

Figure S4. Computed ground state equilibrium structures of lake Baikal cottoid fish rhodopsins. Geometries of A2 retinal chromophore in *C. inermis* (green), *P. jettelesi* (orange), *A. korotneffi* (blue)

and *P. kneri* (red). The relevant bond lengths and backbone dihedral angle are given in Å and degrees.

In order to assess the effect of the first shell of amino acids surrounding the chromophore (subsystem II in Scheme S1), the electrostatic effect of each amino acid in the CASTp cavity and water molecule with at least one atom within 4 Å from any atom of the chromophore, is computed on the basis of our QM/MM models. This is done by turning off the point charges of each residue independently and recomputing the excitation energy (ΔE_{off}). The ΔE-ΔE_{off} value provides an indication of the effect of the residue and reported in Table S4. Accordingly, we start by comparing the electrostatic effects of the amino acids of subsystem II of the *B. taurus* and *P. jettelesi* A2 pigments (i.e. the set template and reference respectively). As shown in Fig. S5 and consistently with previous studies[14,34,35], the *B. taurus* model shows that the largest electrostatic effect is due to the negatively charged putative E113 counterion. E113 is close to the protonated Schiff base and its negative charge must strongly stabilizes the S_0 state with respect to S_1 state leading to a large blue-shift (more than 20 kcal mol^{-1} in the present model). The other sizable but sparse contributions (i.e. larger than 0.5 kcal mol^{-1}) tend to compensate this effect as they are mostly red-shifting. This is consistent with the idea of a substantially "localized" counterion structure in *B. taurus* whose effect is partially quenched by a much more delocalized cloud of cavity residues as also documented in previous QM/MM studies.[14,34,36]

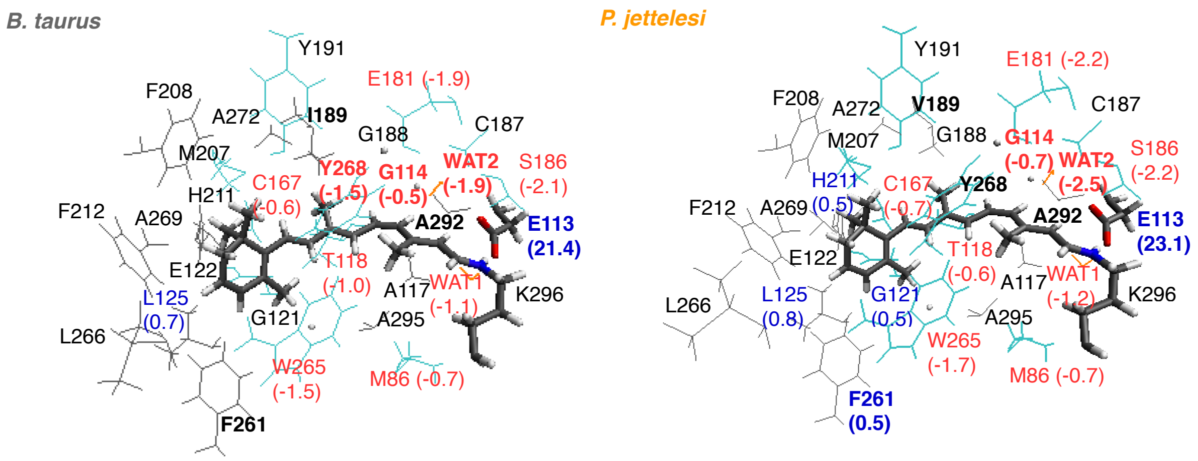

Figure S5. Comparion of subsystem II (i.e. the retinal-binding pocket) of bovine visual pigment (*B. taurus*, left) and the cottoid visual pigment *P. jettelesi* (right). Conventional apolar and polar residues are reported in gray and cyan respectively. Gly residues (hydrogen) are shown as small white spheres. The chromophore and the putative counterion E113 are shown in tube representation. The cavity residues inducing a $\Delta E_{S1\text{-}S0}$ change larger than 0.5 kcal mol^{-1} in absolute value are labelled in red (negative shift) and blue (positive shift) and the corresponding change is given in parenthesis in kcal mol^{-1}. The labels in bold indicate the residues that are not conserved in at least one of the five pigments of the set studied in this report.

Inspection of the electrostatic effects computed for the subsystem II of the reference A2 pigment from *P. jettelesi* shows that these are very similar to those documented for the *B. taurus* pigment. This is consistent with the high level of homology between these proteins with the small root mean square deviation of the position of the atoms in the two cavity subsystems. In fact, notice that the only substitution existing in the chromophore binding pocket is I189V yielding a negligible effect on the

ΔE (it is a replacement of residues without significant change in polarity) and therefore such replacement does not affect the phenotype.

The fact that the results obtained for *B. taurus* modified with the A2 chromophore are in line with those expected when looking at previous studies with the A1 chromophore[37] and, again, with those expected for *P. jettelesi*, suggests that the same electrostatic analysis could be extended to all members of the set. Table S4 shows a complete list of the electrostatic analysis for all the residues in subsystem II for both A1 and A2 chromophores for cottoid fish rhodopsins and the bovine rhodopsin models. It shows that the effect of residues on A2 chromophore is generally similar but larger effect than the one found in A1 chromophore models.

In order to complete the assessment of electrostatic effect of all the different substitutions among species, we have extended our electrostatic analysis to the residues outside the chromophore binding pocket (i.e. to subsystem III in Scheme S1. More specifically, we only assess the subsystem residues that correspond to substitutions across the investigated set. There are a total of 29 residues falling into this category.). In Table S5, we show that there exist residues outside the cavity that have the comparable effect on ΔE as the ones in the cavity and these residues can explain the difference between the two intermediate cases (*C. inermis and P. jettelesi*) as they show the same cavity residues. (Figure S6A).

Figure S6B shows the effect of extra-cavity residues among the four cottoid fish pigments. Between *P. kneri* and *A. korotneffi,* the substitutions of these extra cavity residues has little contribution to the λ_{max} change due to the cancellation of ΔE-ΔE_{off} of opposite signs.

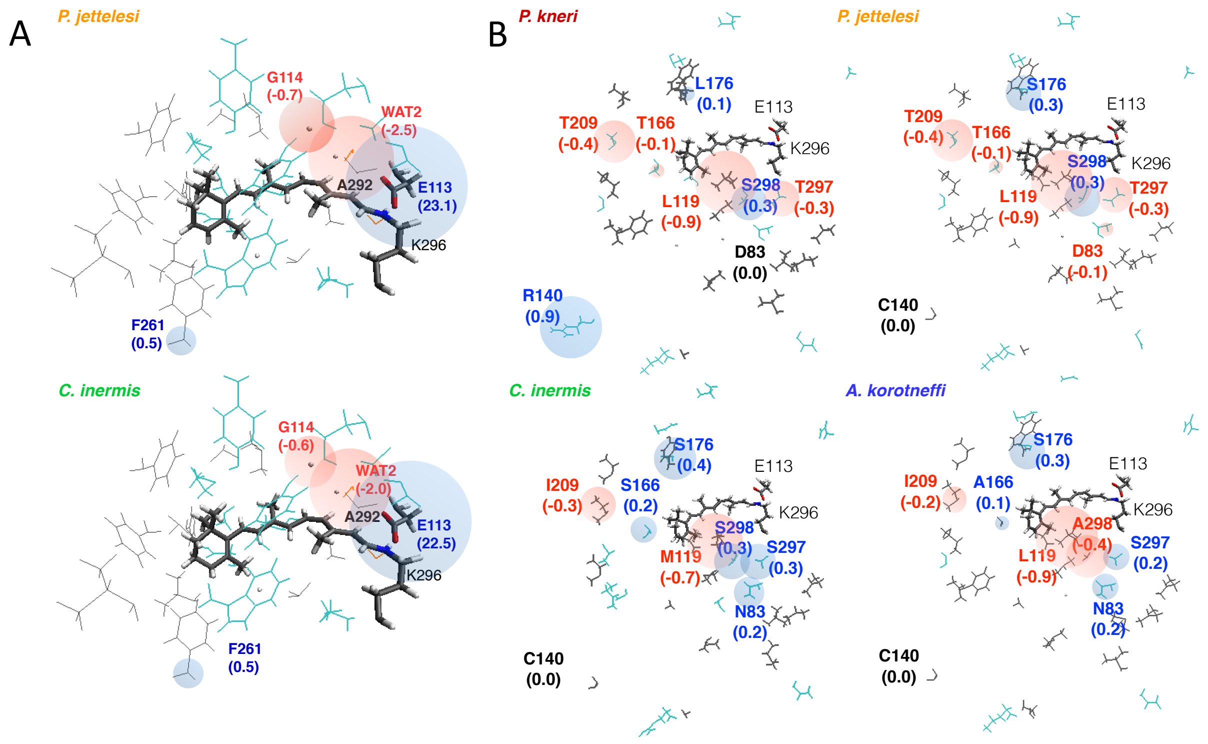

Figure S6. A. Effects of point charges of specific cavity residues between the two intermediate cases: *C. inermis* and *P. jettelesi* with A2 chromophore. Conventional apolar and polar residues are reported in gray and cyan, respectively and Gly residue (hydrogen) is shown as small gray sphere. The chromophore and the putative counterion E113 are shown in tube representation. The labels indicate residues that are not conserved in at least one of the four pigments of the set. $\Delta E\text{-}\Delta E_{off}$ larger than 0.5 kcal mol^{-1} in absolute value are labelled in red (negative shift) and blue (positive shift). The corresponding ΔE change is given in parenthesis in kcal mol^{-1}. B. The same results except with extra-cavity residues for the four cottoid fish pigments with A2 chromophores with the labels showing $\Delta E\text{-}\Delta E_{off}$ larger than 0.2 kcal mol^{-1} in absolute value.

Table S4 Electrostatic effect of the residues in the retinal-binding pocket of cottoid fish rhodopsins and the bovine rhodopsin (*B. taurus*). Number and type of residues are reported in the first column; in case of substitutions the symbols of the residues in each model are reported following the same order of the columns. The shifts caused by every single amino acid sidechain are expressed in kcal mol^{-1}; negative values indicate redshifts and positive values indicate blueshifts.

Residue number and type	*P. kneri*		*P. jettelesi*		*B. taurus*		*C. inermis*		*A. korotneffi*	
	A1	A2	A1	A2	A1	A2	A1	A2	A1	A2
1 WAT	-1.1	-1.3	-1.1	-1.2	-1.1	-1.1	-1.3	-1.4	-1.1	-1.3
**2 WAT**	-1.8	-2.3	-2.2	-2.5	-1.2	-1.9	-1.6	-2.0	-1.4	-1.6
86 M	-0.8	-0.9	-0.7	-0.7	-0.6	-0.7	-0.8	-1.0	-0.7	-0.8
**113 E**	20.3	23.1	19.6	23.1	18.2	21.4	19.6	22.5	19.6	22.9
**114 G-G-G-G-A**	-0.4	-0.8	-0.4	-0.7	-0.6	-0.5	-0.4	-0.6	0.0	0.0
117 A	-0.3	-0.3	-0.2	-0.3	-0.3	-0.2	-0.3	-0.3	-0.3	-0.3
118 T	-1.1	-0.7	-1.1	-0.6	-1.3	-1.0	-1.4	-1.0	-1.2	-0.9
121 G	0.4	0.4	0.4	0.5	0.4	0.4	0.5	0.5	0.4	0.4
122 E	0.2	0.0	0.2	0.1	0.2	0.2	0.2	0.1	0.2	0.1
125 L	0.6	0.7	0.6	0.8	0.6	0.7	0.6	0.8	0.6	0.7
167 C	-0.7	-0.7	-0.7	-0.6	-0.6	-0.6	-0.6	-0.7	-0.7	-0.7
181 E	-2.1	-2.3	-2.0	-2.2	-1.9	-1.9	-2.3	-2.4	-2.1	-2.3
186 S	-2.2	-2.5	-2.0	-2.2	-2.1	-2.1	-2.4	-2.7	-2.1	-2.3
187 C	0.0	-0.1	0.0	-0.1	0.0	-0.1	-0.1	-0.1	0.0	-0.1
188 G	-0.1	-0.5	-0.1	-0.4	0.0	-0.2	0.0	-0.3	0.0	-0.3
189 V-V-I-V-V	0.5	0.3	0.5	0.3	0.5	0.4	0.6	0.4	0.6	0.4
191 Y	0.0	0.3	-0.1	0.1	-0.2	0.0	-0.2	0.1	-0.1	-0.1
207 M	-0.1	0.1	-0.1	0.1	-0.2	0.0	-0.1	0.1	-0.2	0.0
208 F	-0.1	-0.1	0.0	0.0	0.0	0.0	-0.1	-0.1	0.0	-0.1
211 H	0.3	0.5	0.3	0.5	0.2	0.4	0.3	0.4	0.3	0.4
212 F	0.0	0.1	0.1	0.2	0.1	0.1	0.1	0.2	0.1	0.1
**261 Y-F-F-F-F**	-0.6	-0.9	0.3	0.5	0.3	0.4	0.3	0.5	0.3	0.4
265 W	-1.2	-1.4	-1.3	-1.7	-0.5	-1.2	-1.3	-1.4	-1.1	-1.3
266 L	0.2	0.3	0.2	0.3	0.2	0.2	0.2	0.3	0.2	0.2
**268 Y**	-0.4	0.2	-0.5	0.1	-0.5	-1.5	-0.7	-0.2	-0.7	-0.4
269 A	0.0	0.1	0.0	0.1	0.0	0.1	0.0	0.1	0.0	0.0
272 A	0.0	0.0	0.1	0.1	0.0	0.1	0.0	0.0	0.1	0.0
**292 A-A-A-A-S**	0.0	-0.1	0.0	0.0	0.0	0.0	-0.1	-0.1	-0.4	-0.4
295 A	0.0	0.0	0.0	0.0	0.0	0.0	0.0	0.0	0.0	0.0

Table S5 Electrostatic effect of every single point of difference outside the retinal binding pocket of cottoid fish rhodopsins and the bovine rhodopsin (*B. taurus*). Number and type of residues are reported in the first column; all the substitutions the symbols of the residues in each model are reported following the same order of the columns. The shifts caused by every single amino acid sidechain are expressed in kcal mol^{-1}; negative values indicate redshifts and positive values indicate blueshifts.

Residue number and type	*P. kneri*		*P. jettelesi*		*B. taurus*		*C. inermis*		*A. korotneffi*	
	A1	A2	A1	A2	A1	A2	A1	A2	A1	A2
33 S-S-E-N-N	0.1	0.1	0.2	0.1	1.4	1.5	0.1	0.1	0.1	0.1
50 V-V-L-I-I	-0.2	-0.2	-0.2	-0.2	-0.1	-0.2	-0.2	-0.2	-0.2	-0.2
54 V-V-I-V-I	-0.1	-0.2	-0.1	-0.2	0.0	-0.1	-0.1	-0.2	-0.1	-0.1
63 L-L-V-I-L	0.0	-0.1	0.0	-0.1	0.1	0.0	0.0	0.0	0.0	0.0
64 E-D-Q-E-E	0.1	0.1	0.1	0.0	0.1	0.0	0.2	0.1	0.1	0.1
**83 D-D-D-N-N**	0.0	0.0	0.0	-0.1	0.1	0.0	0.2	0.2	0.2	0.2
119 L-L-L-M-L	-0.7	-0.9	-0.7	-0.9	-0.6	-0.7	-0.5	-0.7	-0.7	-0.9
140 R-C-C-C-C	0.7	0.9	0.0	0.0	0.1	0.0	0.0	0.0	0.0	0.0
158 G-A-A-A-A	-0.1	-0.1	-0.1	-0.1	0.0	0.0	-0.1	-0.1	-0.1	-0.1
165 S-L-L-L-L	-0.2	-0.1	-0.1	-0.2	0.0	-0.1	-0.2	-0.1	-0.2	-0.2
166 T-T-A-S-A	-0.1	-0.1	-0.1	-0.1	0.1	0.1	0.2	0.2	0.1	0.1
176 L-S-S-S-S	0.1	0.1	0.3	0.3	0.4	0.3	0.4	0.4	0.4	0.3
200 T-T-N-N-N	-0.2	-0.3	-0.2	-0.3	-0.3	-0.3	-0.3	-0.3	-0.3	-0.3
205 L-L-I-I-I	-0.3	-0.4	-0.3	-0.4	-0.1	-0.2	-0.3	-0.4	-0.3	-0.3
209 T-T-V-I-I	-0.3	-0.5	-0.3	-0.4	0.0	-0.2	-0.2	-0.3	-0.1	-0.2
214 I-I-I-T-I	0.2	0.2	0.2	0.2	0.3	0.1	0.2	0.3	0.2	0.2
217 S-S-I-I-S	0.0	0.1	0.1	0.0	0.2	0.2	0.1	0.1	0.1	0.0
218 V-V-V-I-V	0.1	0.2	0.2	0.1	0.2	0.2	0.2	0.2	0.2	0.2
220 F-F-F-S-F	0.1	0.2	0.1	0.1	0.2	0.1	0.1	0.1	0.1	0.1
234 A-A-A-A-P	0.0	0.0	0.0	-0.1	0.1	0.0	0.0	0.0	0.0	0.0
248 K-K-K-R-K	0.4	0.5	0.4	0.5	0.5	0.5	0.4	0.6	0.4	0.4
256 I-L-I-M-I	-0.1	-0.2	-0.1	-0.2	0.0	-0.1	-0.1	-0.1	-0.1	-0.1
260 G-G-A-S-G	0.0	-0.1	0.0	-0.1	0.1	0.0	0.0	0.0	0.0	0.0
263 V-V-I-M-V	0.0	-0.1	0.0	-0.1	0.1	0.0	-0.1	-0.1	0.0	0.0
274 W-W-Y-Y-W	0.0	0.0	0.1	0.0	0.1	0.0	0.0	0.0	0.0	0.0
282 E-E-D-E-D	0.3	0.4	0.3	0.4	0.5	0.4	0.3	0.4	0.4	0.4
**297 T-T-T-S-S**	-0.2	-0.3	-0.2	-0.3	-0.3	-0.4	0.3	0.3	0.2	0.2
**298 S-S-S-S-A**	0.2	0.3	0.2	0.3	-0.3	-0.4	0.3	0.3	-0.4	-0.4
304 L-L-V-L-M	-0.1	-0.1	0.0	-0.1	0.1	0.0	-0.1	-0.1	-0.1	0.0

4. Electrostatic effect on thermal isomerization barrier

Similar to the ΔE, the contributions of the chromophore on the thermal isomerization barrier is examined by computing the difference in energy for the optimized reactant and transition state *in vacuo* (without the protein, $E_a^T{}_{QM}$). Table S6 shows that the $E_a^T{}_{QM}$ is almost the same throughout all the models (< 1 kcal mol^{-1}) This would mean the difference in thermal isomerization energy barrier is due to the protein.

Table S6. Computed E_a^T values in kcal mol^{-1} for the A1 chromophore of the four selected fish rhodopsins and the template bovine rhodopsin (*B. taurus*) *in vacuo* ($E_a^T{}_{QM}$) and *in zero electrostatic charge for all MM atoms.* ($E_a^T{}_{all\_MM\_off}$)

Condition	*P. kneri*	*P. jettelesi*	*B. taurus*	*C. inermis*	*A. korotneffi*
$E_a^T{}_{QM}$	29.5	29.0	29.0	29.0	29.0
$E_a^T{}_{all\_MM\_off}$	27.1	26.0	25.5	24.6	23.4
$E_a^T{}_{all\_MM\_off}$ - $E_a^T{}_{QM}$	-2.4	-3.0	-3.5	-4.4	-5.6

Unlike the ΔE which is computed with the same ground state equilibrium geometry, the thermal isomerization barrier is computed with two different geometries (reactant and transition state). Hence, the effect of van der Waals interactions may not be the same. To demonstrate the effect of van der Waals interactions as well as the molecular mechanics terms other than the electrostatic on the E_a^T, the E_a^T values are re-computed with all the electrostatic charges on the amino acid residues set to zero ($E_a^T{}_{all\_MM\_off}$, see Table S6). The $E_a^T{}_{all\_MM\_off}$ - $E_a^T{}_{QM}$ values denote the effect of all the molecular mechanics terms (including the van der Walls interactions) other than the electrostatic term on the thermal isomerization barrier. It is interesting to see that the effect of all these terms is going to reduce the barrier and is completely opposite to the trends observed in the full protein models. This would mean that the electrostatic of amino acid residues has a dominating effect on the E_a^T such that it would reverse the trend that is observed in the full protein models.

In order to understand the effect of amino acid residues, a similar point-charge analysis are performed. Instead of computing ΔE_{off}, $E_a^T{}_{off}$ are computed by turning off the point charges of each residue independently and recomputing E_a^T. Table S7 shows the effect of independent amino acid residues in the binding pocket (subsystem II) on the thermal isomerization barrier (E_a^T-$E_a^T{}_{off}$). Figure S7 shows the cavity residue substitutions of G114A from *P. kneri* to *A. korotneffi* has much smaller effect than the other two cavity residues described in the main text (Fig. 3B and 3C) as there is lack of change in polarity. Again, we have extended our electrostatic analysis on the thermal isomerization barrier to the residues outside the chromophore binding pocket (i.e. to subsystem III in Scheme S1. More specifically, we only assess the subsystem residues that correspond to substitutions across the investigated set. There are a total of 29 residues falling into this category.) and these values are reported in Table

S8. However, the effect of extra-cavity residues on the thermal isomerization barrier is much less than that found.

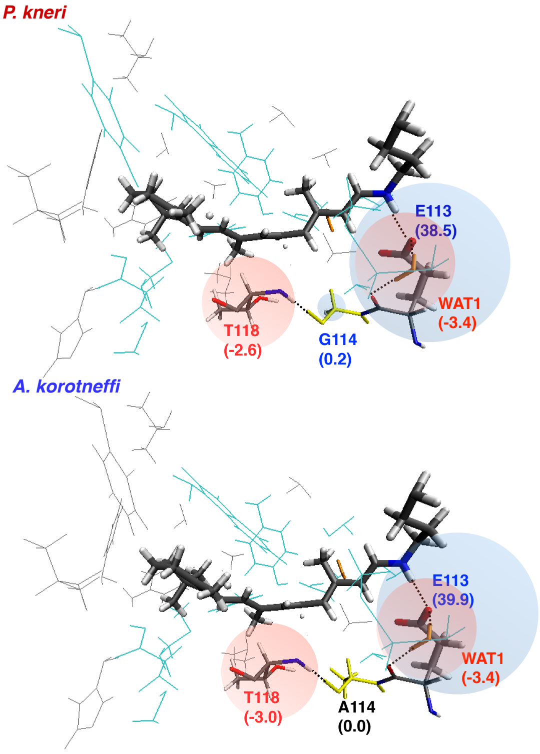

Figure S7. Retinal-binding pockets of transition state of *P. kneri* and *A. korotneffi* with A1 chromophores viewed with substitution (reported in yellow) at residue 114. Conventional apolar and polar residue (side-chain only) are reported in gray and cyan, respectively and glycine residue (hydrogen) is shown as small white sphere. The chromophore, the putative counterion E113 and the full residues which involves in the hydrogen bonding network with the mutated residues are shown in tube representation. ΔE_a^T-$\Delta E_a^T{}_{off}$ larger than 0.5 kcal mol^{-1} in absolute value are labelled in red (negative shift) and blue (positive shift). The corresponding value is given in parenthesis in kcal mol^{-1}.

Table S7 Electrostatic effect of the residues in the retinal-binding pocket of cottoid fish rhodopsins and the bovine rhodopsin (*B. taurus*) on the thermal isomerization barrier. Number and type of residues are reported in the first column; in case of substitutions the symbols of the residues in each model are reported following the same order of the columns. The shifts caused by every single amino acid sidechain are expressed in kcal mol^{-1}; negative values indicate redshifts and positive values indicate blueshifts.

Residue number and type	*P. kneri*	*P. jettelesi*	*B. taurus*	*C. inermis*	*A. korotneffi*
1 WAT	-3.4	-2.5	-2.9	-2.8	-3.4
**2 WAT**	-7.7	-8.7	-4.5	-8.5	-3.0
86 M	-0.1	0.0	0.1	-0.1	0.0
**113 E**	38.5	37.0	32.1	36.7	39.9
**114 G-G-G-G-A**	0.2	0.6	-0.2	0.7	0.0
117 A	-3.0	-2.5	-2.1	-2.6	-2.8
118 T	-2.6	-1.9	-2.2	-1.3	-3.0
121 G	-0.2	1.2	1.5	1.7	1.7
122 E	-0.5	-0.5	0.0	-0.4	-0.5
125 L	1.3	0.9	1.1	1.1	1.1
167 C	-0.5	-0.4	-0.7	-0.8	-0.6
181 E	-3.2	-3.2	-3.5	-4.2	-5.3
186 S	-4.6	-4.2	-4.6	-4.0	-3.3
187 C	1.6	0.9	2.4	1.6	1.7
188 G	2.4	0.1	2.8	2.5	2.9
189 V-V-I-V-V	1.3	2.6	1.9	3.1	1.9
191 Y	-1.2	-1.2	-0.7	-1.3	-1.4
207 M	-1.5	-1.1	-1.1	-1.3	-1.3
208 F	-0.4	-0.4	-0.3	-0.4	-0.5
211 H	-1.4	-0.6	-1.1	-1.0	-1.2
212 F	-0.1	0.2	0.0	0.2	0.0
**261 Y-F-F-F-F**	-2.7	0.1	0.1	0.1	0.0
265 W	-3.0	-3.0	-3.0	-2.8	-3.4
266 L	-0.5	-0.4	-0.5	-0.5	-0.6
**268 Y**	-2.0	-2.5	-1.6	-2.7	-3.4
269 A	0.6	0.4	-0.9	0.5	0.5
272 A	0.6	0.5	0.6	0.7	0.6
**292 A-A-A-A-S**	-0.7	0.8	2.6	2.5	4.1
295 A	-1.6	-1.2	-1.4	-1.5	-1.5

Table S8 Electrostatic effect of the residues in the retinal-binding pocket of cottoid fish rhodopsins and the bovine rhodopsin (*B. taurus*) on the thermal isomerization barrier. Number and type of residues are reported in the first column; in case of substitutions the symbols of the residues in each model are reported following the same order of the columns. The shifts caused by every single amino acid sidechain are expressed in kcal mol^{-1}; negative values indicate redshifts and positive values indicate blueshifts.

Residue number and type	*P. kneri*	*P. jettelesi*	*B. taurus*	*C. inermis*	*A. korotneffi*
**33 S-S-E-N-N**	0.2	0.2	2.2	0.2	0.1
50 V-V-L-I-I	-0.3	-0.2	-0.3	-0.3	-0.3
54 V-V-I-V-I	-0.2	-0.1	-0.2	-0.2	-0.2
63 L-L-V-I-L	0.0	0.0	0.0	0.0	-0.1
64 E-D-Q-E-E	0.1	0.0	0.0	0.2	0.1
**83 D-D-D-N-N**	-0.1	-0.1	-0.1	0.2	0.1
**119 L-L-L-M-L**	-1.4	-1.0	-1.1	-0.8	-1.3
**140 R-C-C-C-C**	1.1	0.0	0.0	0.0	0.0
158 G-A-A-A-A	-0.1	-0.1	-0.1	-0.1	-0.1
165 S-L-L-L-L	-0.2	-0.1	-0.2	-0.2	-0.3
**166 T-T-A-S-A**	-0.1	-0.1	0.0	0.3	0.1
176 L-S-S-S-S	0.2	1.1	0.7	1.3	0.6
200 T-T-N-N-N	-0.4	-0.3	-0.5	-0.5	-0.5
205 L-L-I-I-I	-0.5	-0.4	-0.3	-0.4	-0.5
209 T-T-V-I-I	-0.5	-0.3	-0.2	-0.2	-0.3
214 I-I-I-T-I	0.3	0.2	0.3	0.3	0.2
217 S-S-I-I-S	0.1	0.1	0.2	0.2	0.0
218 V-V-V-I-V	0.3	-0.7	0.2	0.2	0.2
220 F-F-F-S-F	0.2	0.1	0.2	0.2	0.1
234 A-A-A-A-P	0.0	0.0	0.0	0.0	-0.1
248 K-K-K-R-K	0.7	0.5	0.6	0.6	0.6
256 I-L-I-M-I	-0.1	-0.1	-0.1	-0.1	-0.2
260 G-G-A-S-G	0.0	0.0	0.0	0.0	-0.1
263 V-V-I-M-V	0.0	0.0	0.0	-0.1	0.0
274 W-W-Y-Y-W	0.1	0.0	0.0	0.0	0.0
282 E-E-D-E-D	0.5	0.3	0.5	0.4	0.5
**297 T-T-T-S-S**	-0.3	-0.2	-0.6	0.4	0.3
**298 S-S-S-S-A**	0.5	0.5	0.4	0.8	-0.5
304 L-L-V-L-M	0.0	0.0	0.0	-0.1	0.0

5. Mechanisms of extra-cavity substitutions on spectral tuning

Since *C. inermis* presents the same residues of *P. jettelesi* in the cavity, it is needed to account for the effect of extra-cavity substitutions. The main contributor is the substitution T297S which causes a 0.6 kcal mol^{-1} blueshift from *P. jettelesi* to *C. inermis.* In Fig. S8A, it is shown that such substitution would change in orientation of the OH in these sidechains. The hydroxyl oxygen in S297 is closer to the Schiff base than that on T297 and hence stabilize the S_0 state better to give a blueshift. Moreover, the extra methyl group in T297 is destabilizing the S_0 state to give a redshift.

A similar effect can be seen in T166S as well. However, residue 166 is closer to the β-ionone ring of the chromophore and the oxygen in S166 is further away from β-ionone ring than the oxygen in T166. This makes the oxygen in T166 stabilize better the S_1 state to give a red-shift in *P. jettelesi* than *C. inermis.* (Fig. S8B)

Another substitution D83N from *P. jettelesi* to *C. inermis* would cause a blue-shift in ΔE for 0.3 kcal mol^{-1}. This is due to the replacement of nitrogen would cause the carbonyl oxygen to be more negatively charged than the oxygen in the protonated D83. This would make N83 stabilize the Schiff base better than the protonated D83. (Fig. S8B)

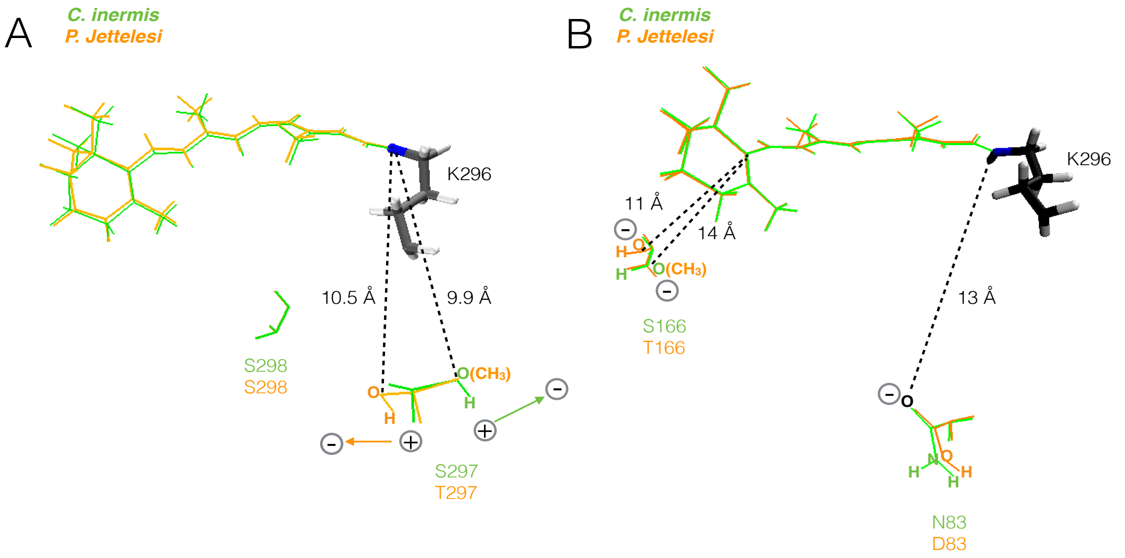

Figure S8 Chromophore with extra-cavity substitution between *P. jettelesi* (yellow) and *C. inermis* (green) with the common retinal-binding lysine 296 is shown tube representation. A. Detailed difference between the T297S substitution. B. Detailed difference between T166S and D83N substitution.

It is worth to note that similar blue shifting effects are computed for *A. korotneffi* , which share the same S297 residue with *C. inermis*. However, *A. korotneffi* displays with respect to *C. inermis* a S298A substitution which would destabilize the S_0 state by changing a partially negative charged hydroxy oxygen in Ser to a partially positive charged hydrogen in Ala (see Fig. S9). This substitution

would cause a red-shift of 0.7 kcal mol^{-1} in *A. korotneffi*. However, this effect is counterbalanced by the other extra-cavity substitutions mentioned above.

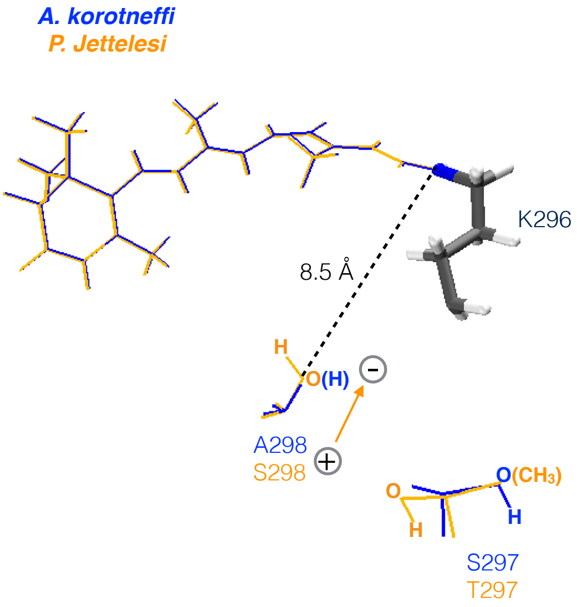

Figure S9. Detailed difference between the S298A and T297S substitutions between *P. jettelesi* (yellow) and *A. korotneffi* (blue).

6. Differences between the computed and experimentally observed E_a^T.

Ala-Laurila et al.[38] proposed that the thermal activation of a complex molecule as rhodopsins follows a non-Arrhenius kinetics described using a $log k = log A(T, E_a^T) - E_a^T/RT$ law. Therefore, according to these authors, while the sign of the slope characterizing the Barlow correlation is controlled by the changes in E_a^T - consistently predicted to be positive in the present work and in ref. 30 - a correct quantitative simulation of the $-log$ k vs. $1/\lambda_{max}$ or the correct fitting of the experimental data to yield the E_a^T value requires a non-Arrhenius model.

The consistency between the E_a^T values produced by our QM/MM models (34-40 kcal mol^{-1}) and the available experimental data has been assessed by assuming that the rate-determining step controlling the thermal noise is the canonical isomerization of the A1 chromophore in agreement with the Luo et al.[39] hypothesis and recent experimental results by Shichida and coworkers.[40] Accordingly, by using the computed E_a^T values and assuming the validity of the Hinshelwood kinetic model originally proposed by Ala-Laurila et al.[38] and adopted in Luo et al.[39] to account for the effect of the chromophore vibrational modes, we calculated 5 A1/A2-bovine rhodopsin rate constant ratios: 1/8.3 (WT), 1/7.3 (A269T), 1/9.3 (F261Y), 1/9.7 (E113D) and 1/301.2 (T118A) to be compared to the measured ratio between A1 Bufo

rhodopsin and A2 Xenopus rhodopsin of 1/8.9. Furthermore, by using the same Hinshelwood model, a quantitative fit to the observed k vs. λ_{max} data for visual pigments could be achieved (Fig. S10). The computed barriers and rate constant ratios are obtained via QM/MM models of the bovine rhodopsin and related mutants and do not contain experimental parameters. The large deviation of T118A from the observed ratio is tentatively assigned to an overestimated barrier for the A1 chromophore.

The use of an Arrhenius model, which is characterized by a constant pre-exponential factor A, for fitting *-log* k vs. 1/T measurements in a restricted temperature range (yielding an acceptable linear fit) may lead to different E_a^T values as a function of the specific range of temperatures considered. Therefore, when using the same assumption that the rate-determining step controlling the thermal noise is the canonical isomerization of the A1 chromophore,[39,40] the reported 22 kcal mol^{-1} activation energy[41] for frog rhodopsin obtained at a body temperature range and the 34-40 kcal mol^{-1} computed in the present work which refer to low temperatures as these are differences in potential energy, may not be inconsistent, They just refer to different values of the *constant pre-exponential factor* or more precisely to *a pre-exponential factor that is temperature dependent* (i.e. to a non-Arrhenius model). Also our computed barrier for TS_{CT} have a few kcal/mol systematic uncertainty on their absolute values due to the MCQC computational error[30] and the fact that we have used a rather stiff protein model (only the cavity residues are relaxed during the calculations). Furthermore, the reactant and transition state structures are computed via conventional geometry optimization of guess structures and correspond to single stationary points on the ground state potential energy surface of the protein. The effect of the protein dynamics at body temperature is thus not incorporated in our calculations but it is assumed to produce a systematic error on the computed E_a^T values leading to limited changes in the computed E_a^T trends.

A first level of complexity introduced by a molecular environment such that of the rhodopsin cavity on the thermal isomerization of the A1 chromophore may be related to the changes in the HBN structure occurring at the reactant (dark adapted) state with respect to the TS_{CT} as a function of the temperature. Such type of changes have been discussed in the past[42]. A model study[43] has shown that the E_a^T value obtained via an Arrhenius plot from low temperature measurements will be larger than the value obtained at an higher range of temperatures if the HBN at the level of the reactant state is more perturbed by the temperature increase than the HBN at the TS_{CT} level[43]. In the language of transition state theory, where the canonical pre-exponential is $(k_BT/h)\exp(\Delta S/R)$, such a situation would impose a temperature dependence on

the ΔS of activation which would decrease as a function of the temperature.

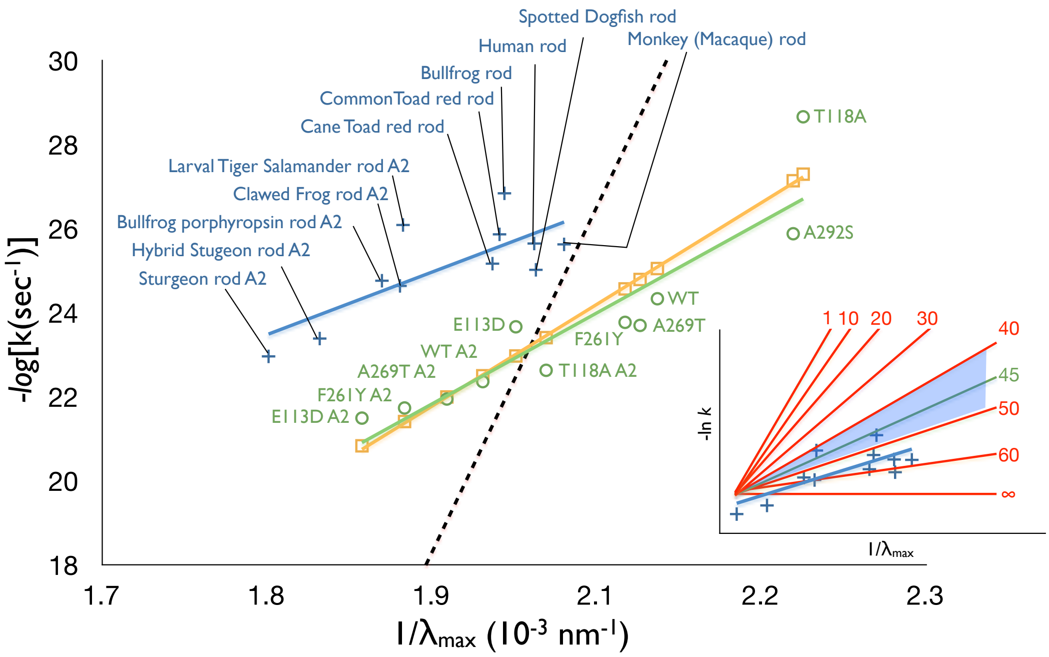

Figure S10. Simulation of the -log$k$ vs. $1/\lambda_{max}$ relationship. By using the set of computed TS_{CT} E_a^T values[30] and the same Hinshelwood pre-exponential factor used in Luo et al. (with m=45)[39] it is possible to successfully simulate the Barlow relation for 11 rod pigments using the *log*k=logA(E_a^T,T)-E_a^T/RT expression (open circles). Those are compared with experimental data (crosses), which are collected in ref. 38. The positive slope of the -*log*k vs. $1/\lambda_{max}$ relation computed with a constant pre-exponential factor (m=1, dashed line) clearly shows that the Hinshelwood pre-exponential factor modulates (decreases) the slope of the relation. Indeed, as shown in the inset (bottom right corner) the slope decreases as a function of the number of modes m but this will never result into a change of the slope from positive to negative. We find that any m value between 40 and 50 reproduces the experimental slope satisfactorily. It is also shown that the empirically derived $E_a^T=0.84hc/\lambda_{max}$ relationship, where the E_a^T values are not computed but obtained via the assumption $E_a^T=E_a^P$ reproduces the experimental slope with m=45 (open squares) and it is substantially parallel to our computed -*log*k vs. $1/\lambda_{max}$ relation. This is promptly explained by the fact that the excited state region near the FC point (determining the value of E_a^P) has the same electronic structure of TS_{CT} (compare Fig. 2C and Fig. 2F in the main text).

7. Cartesian coordinates of the QM subsystem of stationary points reported in this work

Ground state of A1 chromophore of *P. kneri*

```
 C         105.513418    5.449909   -9.800293
 N         104.069586    5.243059   -9.930816
```

H	105.763894	6.434374	-10.164360
H	106.011353	4.704673	-10.400084
H	103.728934	4.281283	-10.033300
C	94.752823	13.313458	-7.765427
C	95.139142	14.632653	-7.076331
C	95.047977	14.543787	-5.561973
C	95.978041	13.457466	-5.036884
C	95.996620	12.185929	-5.862984
C	95.508333	12.130191	-7.124431
C	95.576966	10.904436	-7.969190
C	96.704761	10.217860	-8.251494
C	96.810498	9.047113	-9.138366
C	98.021235	8.429896	-9.234796
C	98.304359	7.219603	-9.994167
C	99.481298	6.600247	-10.292183
C	100.871478	6.992088	-10.030579
C	101.816114	6.014347	-10.206301
C	103.220124	6.213523	-9.966425
C	93.237729	13.072856	-7.626659
C	95.106447	13.453839	-9.256983
C	96.652268	11.028798	-5.140813
C	95.578000	8.636975	-9.907884
C	101.212148	8.411545	-9.622429
H	94.509730	15.433655	-7.455834
H	96.161735	14.883764	-7.354432
H	94.027454	14.331031	-5.262071
H	95.305986	15.499822	-5.111270
H	95.703579	13.211802	-4.017841
H	97.000722	13.832498	-4.984365
H	94.653157	10.599720	-8.430015
H	97.625590	10.547999	-7.800119
H	98.820210	8.851549	-8.656531
H	97.440746	6.676202	-10.327905
H	99.385844	5.638740	-10.767819
H	101.524063	5.013271	-10.487209
H	103.629547	7.195420	-9.807486
H	92.934668	12.966978	-6.593599
H	92.676636	13.898376	-8.057528
H	92.930476	12.169261	-8.142740
H	94.795548	12.595212	-9.841335
H	94.613176	14.326744	-9.676689
H	96.176927	13.577735	-9.398612
H	96.270650	10.977874	-4.123172
H	96.474687	10.074724	-5.616465
H	97.727060	11.172401	-5.064985
H	95.217692	9.465350	-10.508478
H	95.754778	7.806376	-10.574819
H	94.778945	8.349267	-9.234461
H	101.042737	8.554879	-8.560979
H	102.243798	8.676334	-9.812499
H	100.584767	9.120307	-10.147128
H	105.862558	5.359691	-8.750488

Ground state of A2 chromophore of *P. kneri*

C	105.538798	5.448030	-9.806164
N	104.093497	5.252698	-9.945220
H	105.801263	6.425724	-10.179617
H	106.034344	4.691802	-10.392779
H	103.748174	4.292294	-10.044853

C	94.742439	13.349054	-7.795419
C	95.243769	14.680333	-7.205352
C	95.478525	14.620797	-5.724096
C	95.833996	13.466675	-5.142136
C	95.965274	12.211250	-5.910703
C	95.520586	12.156878	-7.193112
C	95.610866	10.947447	-8.048627
C	96.725034	10.211044	-8.251374
C	96.840371	9.047518	-9.145485
C	98.045389	8.416799	-9.219743
C	98.328348	7.211945	-9.988483
C	99.506597	6.599472	-10.292770
C	100.894460	6.995261	-10.025595
C	101.842859	6.024271	-10.213444
C	103.246372	6.225697	-9.973175
C	93.248100	13.167636	-7.467736
C	94.923292	13.444225	-9.320372
C	96.581705	11.060313	-5.145593
C	95.625661	8.656232	-9.953970
C	101.231734	8.415647	-9.615723
H	94.530339	15.462939	-7.447795
H	96.177833	14.956712	-7.694720
H	95.414311	15.528959	-5.150435
H	96.031369	13.425958	-4.088336
H	94.710624	10.681789	-8.574870
H	97.626223	10.497389	-7.736022
H	98.836979	8.818088	-8.617122
H	97.465087	6.669643	-10.324967
H	99.415033	5.639515	-10.772368
H	101.553792	5.022271	-10.494377
H	103.656190	7.207010	-9.811135
H	93.069561	13.185840	-6.400108
H	92.658436	13.960215	-7.921429
H	92.873288	12.222645	-7.848285
H	94.472376	12.609181	-9.844032
H	94.448629	14.349245	-9.690132
H	95.972998	13.483474	-9.596774
H	96.167315	11.038943	-4.140241
H	96.392489	10.103314	-5.611939
H	97.656338	11.184662	-5.041458
H	95.263733	9.504917	-10.523625
H	95.829575	7.861130	-10.655735
H	94.820640	8.325473	-9.307490
H	101.083035	8.552484	-8.550170
H	102.257418	8.688499	-9.825973
H	100.589148	9.122100	-10.124460
H	105.881525	5.364956	-8.753352

Ground state of A1 chromophore of *P. jettelesi*

C	105.537558	5.503758	-9.836843
N	104.097126	5.281859	-9.978979
H	105.773243	6.498988	-10.181142
H	106.048959	4.778378	-10.450009
H	103.767334	4.315973	-10.063841
C	94.969866	13.323914	-7.384674
C	95.322799	14.518035	-6.478487
C	95.095509	14.217101	-5.003914
C	95.985872	13.059603	-4.569923

C	96.003599	11.902034	-5.547135
C	95.598698	12.027826	-6.832488
C	95.668635	10.892709	-7.796813
C	96.793453	10.206149	-8.091168
C	96.906469	9.075506	-9.025025
C	98.098512	8.424409	-9.113066
C	98.335085	7.225175	-9.909917
C	99.492115	6.604715	-10.270002
C	100.888188	7.003904	-10.061972
C	101.835846	6.034859	-10.267703
C	103.236027	6.242491	-10.011460
C	93.439046	13.160389	-7.465842
C	95.506236	13.634130	-8.794484
C	96.564414	10.635152	-4.944290
C	95.690344	8.681471	-9.825743
C	101.233792	8.431517	-9.684114
H	94.747124	15.386510	-6.789216
H	96.372482	14.771847	-6.621392
H	94.054217	13.967116	-4.828836
H	95.309113	15.098435	-4.401780
H	95.667892	12.695805	-3.595603
H	97.011742	13.403707	-4.436329
H	94.750926	10.644828	-8.301787
H	97.700473	10.484848	-7.581711
H	98.904112	8.793787	-8.508016
H	97.450517	6.696202	-10.211515
H	99.375731	5.652070	-10.758071
H	101.548809	5.028448	-10.536039
H	103.633797	7.228070	-9.849917
H	93.159275	12.359506	-8.142488
H	93.001021	12.936677	-6.501552
H	92.980019	14.072850	-7.837778
H	95.224290	12.880024	-9.520720
H	95.109092	14.583449	-9.143716
H	96.591031	13.706543	-8.795766
H	97.639070	10.721164	-4.800925
H	96.122439	10.473856	-3.963369
H	96.373963	9.755722	-5.541560
H	95.950023	8.098399	-10.698132
H	95.000252	8.094697	-9.228562
H	95.162256	9.557461	-10.180278
H	102.235910	8.716401	-9.977851
H	100.543877	9.124284	-10.146975
H	101.169143	8.575771	-8.611328
H	105.884586	5.398529	-8.787679

Ground state of A2 chromophore of *P. jettelesi*

C	105.527808	5.509205	-9.841787
N	104.086560	5.290675	-9.988102
H	105.768622	6.504081	-10.183808
H	106.037424	4.782695	-10.455143
H	103.759896	4.324121	-10.084064
C	94.881539	13.338067	-7.455915
C	95.426389	14.534934	-6.649639
C	95.529753	14.257928	-5.175110
C	95.772672	13.012319	-4.744908
C	95.903604	11.874968	-5.677016
C	95.541410	12.024986	-6.977478
C	95.623235	10.922254	-7.969059
C	96.727836	10.181032	-8.201778

C	96.849602	9.070765	-9.158759
C	98.037423	8.407915	-9.214184
C	98.308506	7.217738	-10.011276
C	99.482160	6.602526	-10.325182
C	100.869523	7.001396	-10.066238
C	101.825988	6.042731	-10.282086
C	103.224032	6.250733	-10.014477
C	93.357470	13.228082	-7.260895
C	95.178174	13.616338	-8.940894
C	96.442074	10.607805	-5.052031
C	95.655963	8.739730	-10.022532
C	101.199156	8.421105	-9.647648
H	94.794989	15.399937	-6.832537
H	96.418157	14.796718	-7.020586
H	95.471147	15.081216	-4.482735
H	95.893984	12.805402	-3.695735
H	94.733427	10.740961	-8.547318
H	97.612043	10.401546	-7.628421
H	98.817473	8.758498	-8.566187
H	97.440337	6.686405	-10.353141
H	99.387665	5.649486	-10.816287
H	101.544589	5.039107	-10.566741
H	103.619380	7.235538	-9.843744
H	92.952954	12.380574	-7.805522
H	93.096601	13.106628	-6.217305
H	92.859307	14.121472	-7.628725
H	94.719055	12.887074	-9.598370
H	94.787934	14.591248	-9.219390
H	96.246084	13.616712	-9.139959
H	97.518234	10.672545	-4.912864
H	95.997673	10.472233	-4.068928
H	96.228077	9.725267	-5.637550
H	95.893285	8.023129	-10.795680
H	94.845001	8.334112	-9.427191
H	95.287524	9.631478	-10.516508
H	102.216652	8.708786	-9.877862
H	100.536091	9.124135	-10.134160
H	101.073234	8.545544	-8.577850
H	105.871994	5.400380	-8.791463

Ground state of A1 chromophore of *B. taurus*

C	46.770241	12.318034	15.140922
N	45.789857	12.589629	16.194431
H	46.847483	11.251573	14.997518
H	47.724419	12.708263	15.457444
H	45.896955	13.451805	16.737447
C	34.800879	6.959016	17.758763
C	34.237407	6.180557	16.555699
C	33.348233	7.039428	15.668267
C	34.149508	8.207481	15.106943
C	35.036302	8.894153	16.126063
C	35.385354	8.310800	17.297575
C	36.295920	8.973130	18.273327
C	37.518754	9.465298	17.979303
C	38.437146	10.139033	18.911472
C	39.633786	10.573358	18.425712
C	40.641626	11.301881	19.186475
C	41.931947	11.622337	18.889214

C	42.771720	11.257990	17.742362
C	43.919616	11.987810	17.572959
C	44.835762	11.770645	16.488564
C	33.686768	7.204131	18.796902
C	35.889726	6.085357	18.408599
C	35.520519	10.248964	15.659834
C	38.024867	10.265542	20.357250
C	42.375571	10.110804	16.834942
H	33.697560	5.307776	16.914855
H	35.068124	5.810628	15.955902
H	32.501023	7.411425	16.235722
H	32.938164	6.444137	14.853940
H	33.472677	8.943742	14.678748
H	34.778936	7.870146	14.282935
H	35.938268	9.020863	19.286522
H	37.868515	9.370618	16.965102
H	39.826627	10.390203	17.386024
H	40.304961	11.706306	20.121790
H	42.417381	12.279405	19.591437
H	44.156551	12.803654	18.240059
H	44.762022	10.902271	15.858805
H	33.255536	6.259569	19.118944
H	34.070699	7.700985	19.681407
H	32.886943	7.818753	18.402852
H	36.283071	6.522238	19.319109
H	35.481459	5.111783	18.666669
H	36.724219	5.926669	17.730535
H	36.292980	10.150113	14.900786
H	34.693449	10.790385	15.204574
H	35.914779	10.857300	16.460638
H	36.969971	10.489718	20.441465
H	38.207886	9.332764	20.883761
H	38.545853	11.059146	20.871120
H	41.921556	9.314331	17.410033
H	41.644729	10.434916	16.102144
H	43.208910	9.692788	16.286266
H	46.495812	12.786544	14.171679

Ground state of A2 chromophore of *B. taurus*

C	46.725369	12.300738	15.165561
N	45.744561	12.553906	16.222906
H	46.802814	11.236801	15.005118
H	47.676663	12.685496	15.496040
H	45.837856	13.416936	16.766733
C	34.718867	6.922985	17.871020
C	34.351854	6.038360	16.662190
C	33.685655	6.800367	15.550750
C	33.984396	8.092023	15.355263
C	34.931661	8.817878	16.222538
C	35.339258	8.255075	17.389373
C	36.272720	8.925370	18.328129
C	37.469748	9.455336	17.994526
C	38.401534	10.114815	18.924089
C	39.583101	10.579833	18.434160
C	40.584025	11.317957	19.198101
C	41.875639	11.631208	18.902755
C	42.720205	11.238832	17.769482
C	43.884204	11.944029	17.606749
C	44.786850	11.736013	16.508921

C	33.453125	7.231165	18.694733
C	35.702340	6.119965	18.741346
C	35.355122	10.173200	15.702613
C	37.998150	10.187679	20.374944
C	42.313662	10.093100	16.863910
H	33.717066	5.223102	16.998604
H	35.258315	5.579098	16.266373
H	33.021400	6.277297	14.883454
H	33.548477	8.640768	14.539339
H	35.960393	8.945110	19.357371
H	37.788674	9.392434	16.967854
H	39.761372	10.431104	17.386083
H	40.235443	11.745950	20.118553
H	42.361066	12.294707	19.597984
H	44.131491	12.757157	18.273870
H	44.703861	10.876048	15.868719
H	32.701909	7.733720	18.098059
H	33.016015	6.313952	19.081074
H	33.681653	7.867836	19.543332
H	35.913337	6.606502	19.686170
H	35.282377	5.144273	18.970350
H	36.647163	5.962262	18.229104
H	35.761621	10.807874	16.476706
H	36.101755	10.073103	14.918500
H	34.496015	10.680888	15.269468
H	37.032079	10.665595	20.483611
H	37.919400	9.189880	20.794977
H	38.695887	10.740656	20.983814
H	41.614342	10.427905	16.105648
H	43.150212	9.644376	16.344618
H	41.822149	9.317189	17.435795
H	46.454786	12.787572	14.203810

Ground state of A1 chromophore of *C. inermis*

C	105.638677	5.435439	-9.821542
N	104.196971	5.214613	-9.955288
H	105.879580	6.414278	-10.205429
H	106.152254	4.687684	-10.404328
H	103.861404	4.252294	-10.063409
C	95.021637	13.292621	-7.425123
C	95.358751	14.507906	-6.541363
C	95.137503	14.232324	-5.061074
C	96.043420	13.094711	-4.607415
C	96.072123	11.917434	-5.561578
C	95.666134	12.013803	-6.849521
C	95.738694	10.863927	-7.795847
C	96.854670	10.151794	-8.064694
C	96.990932	9.024424	-8.999799
C	98.187209	8.375263	-9.055834
C	98.439173	7.204901	-9.888794
C	99.598108	6.578834	-10.234406
C	100.994797	6.949024	-9.974460
C	101.933114	5.972902	-10.181072
C	103.339333	6.177720	-9.960691
C	93.492449	13.109137	-7.501944
C	95.553279	13.585359	-8.840579
C	96.638581	10.666454	-4.931548
C	95.835293	8.616190	-9.888455
C	101.346193	8.356700	-9.535891

H	94.770248	15.361663	-6.867731
H	96.404204	14.774631	-6.691314
H	94.099689	13.972846	-4.879004
H	95.340547	15.127330	-4.476178
H	95.733301	12.745200	-3.625432
H	97.065175	13.454025	-4.482495
H	94.824248	10.633555	-8.312709
H	97.756882	10.425301	-7.543513
H	98.974416	8.724824	-8.415635
H	97.556202	6.714224	-10.255261
H	99.482188	5.645916	-10.760734
H	101.638253	4.976762	-10.477838
H	103.742239	7.160795	-9.792236
H	93.059352	12.882154	-6.535974
H	93.021223	14.014931	-7.874424
H	93.219648	12.304478	-8.176960
H	96.636475	13.678169	-8.843383
H	95.286153	12.812999	-9.553191
H	95.139393	14.520618	-9.207594
H	96.440083	9.772380	-5.504645
H	97.714639	10.751460	-4.797730
H	96.204704	10.532010	-3.943043
H	94.993919	9.286328	-9.797593
H	96.139664	8.630528	-10.930451
H	95.482661	7.618973	-9.650962
H	100.694415	9.080211	-10.007995
H	101.220326	8.460317	-8.463634
H	102.367496	8.634927	-9.760256
H	105.981445	5.372441	-8.767608

Ground state of A2 chromophore of *C. inermis*

C	105.670025	5.437650	-9.825071
N	104.227445	5.232936	-9.974861
H	105.927845	6.411765	-10.210153
H	106.180404	4.680559	-10.398483
H	103.883253	4.271763	-10.064853
C	94.976894	13.309950	-7.430698
C	95.450179	14.527452	-6.609312
C	95.512129	14.259583	-5.131809
C	95.764473	13.022421	-4.683650
C	95.962148	11.883471	-5.600966
C	95.645002	12.015014	-6.914800
C	95.768720	10.905164	-7.891990
C	96.863193	10.132457	-8.069176
C	97.017606	9.026122	-9.025793
C	98.208390	8.366574	-9.063742
C	98.466489	7.196396	-9.896084
C	99.630391	6.578525	-10.237277
C	101.023589	6.960329	-9.976169
C	101.968617	5.993938	-10.194631
C	103.374123	6.200414	-9.972544
C	93.448985	13.155765	-7.304175
C	95.331789	13.589651	-8.902552
C	96.474964	10.623011	-4.944195
C	95.872666	8.636383	-9.936629
C	101.366095	8.368165	-9.531006
H	94.791881	15.366955	-6.814298
H	96.442667	14.827083	-6.947459
H	95.401570	15.082591	-4.446453

H	95.851426	12.826888	-3.629315
H	94.905987	10.745399	-8.512961
H	97.726775	10.337463	-7.459588
H	98.984149	8.699644	-8.401272
H	97.587020	6.697871	-10.260564
H	99.523622	5.642030	-10.759176
H	101.676905	4.995319	-10.486039
H	103.780008	7.183401	-9.809797
H	93.144388	13.023346	-6.273741
H	92.941969	14.034264	-7.694723
H	93.094505	12.297930	-7.867155
H	96.407381	13.623215	-9.052178
H	94.925523	12.845966	-9.578275
H	94.925385	14.551408	-9.202572
H	96.258764	9.733058	-5.518160
H	97.549018	10.673581	-4.785256
H	96.010692	10.512808	-3.967390
H	95.123580	9.411122	-10.003901
H	96.230475	8.458096	-10.944461
H	95.380550	7.735169	-9.584887
H	100.713771	9.092103	-10.002013
H	101.234251	8.465693	-8.459057
H	102.388252	8.649888	-9.747231
H	106.000172	5.374004	-8.766876

Ground state of A1 chromophore of *A. korotneffi*

C	105.463522	5.428977	-9.784237
N	104.021395	5.209300	-9.926740
H	105.708847	6.417066	-10.143798
H	105.971130	4.691065	-10.384140
H	103.698072	4.244744	-10.054659
C	94.889375	13.301092	-7.392806
C	95.254454	14.476943	-6.467478
C	95.044919	14.151269	-4.995647
C	95.922338	12.972219	-4.591479
C	95.933663	11.836986	-5.596260
C	95.520176	11.990344	-6.876401
C	95.590282	10.883382	-7.873379
C	96.703193	10.173090	-8.155657
C	96.810646	9.063348	-9.117196
C	97.996752	8.399432	-9.192852
C	98.260949	7.209909	-9.994656
C	99.429124	6.577534	-10.294450
C	100.820933	6.955707	-10.027499
C	101.762940	5.976940	-10.210830
C	103.157495	6.168878	-9.927691
C	93.356896	13.147323	-7.461178
C	95.411759	13.642970	-8.801401
C	96.496033	10.556200	-5.019875
C	95.602813	8.742883	-9.964076
C	101.165156	8.367973	-9.597663
H	94.671343	15.348886	-6.753077
H	96.301037	14.736332	-6.621300
H	94.002416	13.914869	-4.810604
H	95.280140	15.019252	-4.382623
H	95.596502	12.587435	-3.628071
H	96.951297	13.301430	-4.445234
H	94.685983	10.682517	-8.421730
H	97.603431	10.408709	-7.613541

H	98.786097	8.752857	-8.557958
H	97.390043	6.689871	-10.346833
H	99.328294	5.635915	-10.804998
H	101.472267	4.984783	-10.521903
H	103.553965	7.147978	-9.727906
H	93.066502	12.354913	-8.142834
H	92.925505	12.918284	-6.495236
H	92.902128	14.066603	-7.821347
H	95.102147	12.918406	-9.545839
H	95.031105	14.611710	-9.113372
H	96.497457	13.691641	-8.816983
H	96.279486	9.686145	-5.622057
H	97.574675	10.626984	-4.899256
H	96.074359	10.389954	-4.030879
H	94.765440	8.434761	-9.347733
H	95.295065	9.618012	-10.526424
H	95.792721	7.954247	-10.676745
H	101.012341	8.493565	-8.531464
H	102.192711	8.640347	-9.801915
H	100.527569	9.082595	-10.101375
H	105.812222	5.337725	-8.734647

Ground state of A2 chromophore of *A. korotneffi*

C	105.497170	5.438902	-9.797579
N	104.055237	5.228895	-9.954052
H	105.753132	6.425516	-10.152930
H	106.006817	4.697767	-10.392061
H	103.726596	4.266243	-10.080879
C	94.886111	13.332568	-7.420019
C	95.379112	14.519083	-6.565565
C	95.447215	14.205890	-5.096791
C	95.699246	12.954388	-4.689344
C	95.864646	11.838639	-5.641327
C	95.541004	12.014561	-6.948761
C	95.643066	10.929624	-7.957427
C	96.737387	10.166943	-8.168384
C	96.855925	9.069446	-9.141221
C	98.034655	8.391215	-9.197269
C	98.300218	7.213979	-10.017195
C	99.469509	6.594040	-10.335114
C	100.859337	6.974552	-10.062948
C	101.804522	6.003549	-10.268623
C	103.198414	6.194300	-9.977962
C	93.357024	13.196189	-7.289521
C	95.237018	13.650562	-8.885143
C	96.384476	10.557493	-5.028222
C	95.667608	8.778439	-10.026478
C	101.200707	8.384486	-9.622596
H	94.730809	15.372874	-6.742366
H	96.373829	14.816382	-6.900153
H	95.355804	15.010182	-4.386260
H	95.788228	12.725001	-3.641558
H	94.768117	10.770878	-8.564014
H	97.611854	10.358695	-7.570099
H	98.812504	8.718563	-8.533563
H	97.430064	6.697599	-10.376539
H	99.371807	5.657508	-10.855151
H	101.513824	5.010187	-10.576992
H	103.597750	7.172971	-9.781025
H	92.986848	12.361572	-7.876798

H	93.056668	13.036694	-6.261634
H	92.861012	14.094534	-7.648151
H	94.819802	12.929270	-9.578265
H	94.839683	14.624708	-9.156649
H	96.312243	13.675925	-9.039429
H	96.176183	9.686923	-5.633495
H	97.458245	10.610240	-4.866063
H	95.919484	10.407849	-4.056488
H	94.814258	8.459518	-9.437810
H	95.378089	9.669473	-10.572896
H	95.873296	8.008769	-10.755785
H	101.080490	8.493366	-8.550144
H	102.218980	8.669058	-9.855113
H	100.542204	9.100178	-10.096848
H	105.833005	5.344308	-8.743560

TS_{CT} of A1 of *P. kneri*

C	105.686514	5.463148	-9.889179
N	104.294558	5.241249	-10.166725
H	105.981293	6.442239	-10.246253
H	106.271715	4.726630	-10.426187
H	103.949002	4.294232	-10.143756
C	94.681872	13.280002	-7.614576
C	95.013660	14.562277	-6.831841
C	94.818401	14.396264	-5.335391
C	95.699170	13.273268	-4.804512
C	95.876980	12.076780	-5.709609
C	95.472910	12.084855	-7.021319
C	95.670121	10.978754	-7.926142
C	96.634388	9.985824	-7.938002
C	96.818903	9.132832	-9.040071
C	97.943043	8.238405	-8.993116
C	98.565189	7.740001	-10.095126
C	99.726851	6.827783	-10.080243
C	101.046508	7.154729	-10.069690
C	102.031329	6.075640	-10.159047
C	103.371375	6.245881	-10.035449
C	93.176144	12.975995	-7.529336
C	95.066801	13.550678	-9.084359
C	96.553471	10.919315	-5.010422
C	95.892702	9.152185	-10.231484
C	101.521624	8.592200	-9.987237
H	94.396639	15.371133	-7.209878
H	96.047092	14.839986	-7.031849
H	93.777402	14.189108	-5.114884
H	95.072154	15.318720	-4.821770
H	95.319683	12.932058	-3.848498
H	96.704978	13.639455	-4.597491
H	95.009729	10.982840	-8.770073
H	97.363277	9.926193	-7.156577
H	98.365382	8.040900	-8.024219
H	98.206106	8.038131	-11.065750
H	99.457397	5.784651	-10.135114
H	101.661920	5.074689	-10.321863
H	103.796302	7.220136	-9.860393
H	92.847527	12.801385	-6.514043
H	92.600615	13.805161	-7.928692
H	92.914952	12.093319	-8.105308
H	94.716462	12.788018	-9.771140

H	94.617414	14.483135	-9.408030
H	96.142759	13.646086	-9.204217
H	96.363327	10.992562	-3.947785
H	96.196320	9.954119	-5.344330
H	97.631943	10.959463	-5.140830
H	95.878131	10.122347	-10.713267
H	96.179646	8.414623	-10.960378
H	94.885836	8.913425	-9.908503
H	102.027402	8.785480	-9.047828
H	102.251513	8.809617	-10.761220
H	100.710355	9.302507	-10.085430
H	105.963912	5.394131	-8.809641

TS_{CT} of A1 of *A. korotneffi*

C	105.730784	5.473975	-9.878654
N	104.340385	5.241827	-10.148218
H	106.016456	6.458762	-10.225388
H	106.325945	4.748604	-10.419417
H	103.996870	4.294509	-10.157567
C	94.991205	13.156270	-7.412807
C	95.335253	14.286614	-6.424894
C	95.104704	13.895072	-4.974093
C	95.978580	12.700859	-4.610861
C	96.043014	11.616368	-5.666131
C	95.660548	11.833586	-6.959874
C	95.791871	10.827825	-8.007602
C	96.762532	9.862359	-8.147707
C	96.905826	9.053297	-9.300845
C	98.002592	8.134952	-9.320461
C	98.645665	7.732681	-10.454967
C	99.794867	6.809526	-10.471950
C	101.112471	7.144210	-10.421454
C	102.104947	6.076409	-10.459865
C	103.417856	6.247772	-10.153378
C	93.464748	12.960912	-7.486471
C	95.509408	13.603525	-8.795097
C	96.589237	10.316488	-5.125402
C	95.947750	9.137005	-10.456558
C	101.567775	8.582077	-10.257178
H	94.747558	15.162568	-6.682076
H	96.381021	14.560403	-6.552926
H	94.060668	13.654436	-4.807753
H	95.338559	14.730227	-4.319654
H	95.637860	12.262716	-3.678192
H	97.005107	13.021678	-4.431787
H	95.079254	10.910631	-8.806058
H	97.529229	9.770530	-7.405119
H	98.422379	7.859479	-8.369964
H	98.318455	8.132339	-11.399674
H	99.528778	5.769623	-10.585436
H	101.758663	5.076863	-10.676453
H	103.813274	7.217368	-9.900410
H	93.191500	12.191930	-8.202813
H	93.043538	12.675090	-6.531891
H	92.984517	13.881455	-7.803901
H	95.200744	12.947377	-9.601968
H	95.117466	14.587879	-9.028442
H	96.594101	13.669471	-8.807222
H	96.258189	9.444026	-5.670261

H	97.676323	10.333494	-5.115116
H	96.262436	10.197847	-4.098554
H	94.992684	8.732450	-10.136396
H	95.798175	10.152580	-10.797690
H	96.286230	8.541688	-11.287822
H	101.988035	8.754170	-9.272033
H	102.360334	8.822311	-10.960376
H	100.764457	9.293151	-10.406154
H	106.009827	5.401252	-8.800873

References

1.Thompson, J. D., Higgins, D. G. and Gibson, T. J. CLUSTAL W: improving the sensitivity of progressive multiple sequence alignment through sequence weighting, position-specific gap penalties and weight matrix choice. *Nucleic Acids Res* **22,** 4673-80 (1994).

2.Okada, T., Sugihara, M., Bondar, A. N., Elstner, M., Entel, P. and Buss, V. The retinal conformation and its environment in rhodopsin in light of a new 2.2 A crystal structure. *J Mol Biol* **342,** 571-83 (2004).doi:10.1016/j.jmb.2004.07.044

3.Dunbrack, R. L. and Karplus, M. Backbone-dependent rotamer library for proteins. Application to side-chain prediction. *J Mol Biol* **230,** 543-74 (1993).doi:10.1006/jmbi.1993.1170

4.Ponder, J. W. and Richards, F. M. Tertiary templates for proteins. Use of packing criteria in the enumeration of allowed sequences for different structural classes. *Journal of Molecular Biology* **193,** 775-91 (1987).

5.Sutcliffe, M. J., Hayes, F. R. and Blundell, T. L. Knowledge based modelling of homologous proteins, Part II: Rules for the conformations of substituted sidechains. *Protein Engineering* **1,** 385-92 (1987).

6.Accelrys Software Inc. QUANTA Modeling Environment. (2006).

7.Li, H., Robertson, A. D. and Jensen, J. H. Very fast empirical prediction and rationalization of protein pKa values. *Proteins: Structure, Function, and Bioinformatics* **61,** 704-721 (2005).

8.Olsson, M. H., Søndergaard, C. R., Rostkowski, M. and Jensen, J. H. PROPKA3: consistent treatment of internal and surface residues in empirical p K a predictions. *Journal of Chemical Theory and Computation* **7,** 525-537 (2011).doi:ct100578z

9.Fahmy, K., Jäger, F., Beck, M., Zvyaga, T. A., Sakmar, T. P. and Siebert, F. Protonation states of membrane-embedded carboxylic acid groups in rhodopsin and metarhodopsin II: a Fourier-transform infrared spectroscopy study of site-directed mutants. *Proc Natl Acad Sci U S A* **90,** 10206-10 (1993).

10.Yan, E. C., Kazmi, M. A., Ganim, Z., Hou, J. -M., Pan, D., Chang, B. S., Sakmar, T. P. and Mathies, R. A. Retinal counterion switch in the photoactivation of the G protein-coupled receptor rhodopsin. *Proceedings of the National Academy of Sciences* **100,** 9262-9267 (2003).

11.Sekharan, S. and Buss, V. Glutamic acid 181 is uncharged in dark-adapted visual rhodopsin. *Journal of the American Chemical Society* **130,** 17220-17221 (2008).

12.Frähmcke, J. S., Wanko, M., Phatak, P., Mroginski, M. A. and Elstner, M. The protonation state of Glu181 in rhodopsin revisited: interpretation of experimental data on the basis of QM/MM calculations. *J Phys Chem B* **114,** 11338-52 (2010).doi:10.1021/jp104537w

13.Sandberg, M. N., Amora, T. L., Ramos, L. S., Chen, M. H., Knox, B. E. and Birge, R. R. Glutamic acid 181 is negatively charged in the bathorhodopsin photointermediate of visual rhodopsin. *J Am Chem Soc* **133,** 2808-11 (2011).doi:10.1021/ja1094183

14.Tomasello, G., Olaso-González, G., Altoè, P., Stenta, M., Serrano-Andrés, L., Merchán, M., Orlandi, G., Bottoni, A. and Garavelli, M. Electrostatic control of the photoisomerization efficiency and optical properties in visual pigments: on the role of counterion quenching. *J Am Chem Soc* **131,** 5172-86 (2009).doi:10.1021/ja808424b

15.Roos, B. O., Bruna, P., Peyerimhoff, S. D., Shepard, R., Cooper, D. L., Gerratt, J. and Raimondi, M. Ab Initio Methods in Quantum Chemistry, II. *Advances in Chemical Physics* **69,** 399-446 (1987).

16.Cornell, W. D., Cieplak, P., Bayly, C. I., Gould, I. R., Merz, K. M., Ferguson, D. M., Spellmeyer, D. C., Fox, T., Caldwell, J. W. and Kollman, P. A. A second generation force field for the simulation of proteins, nucleic acids, and organic molecules. *Journal of the American Chemical Society* **117,** 5179-5197 (1995).

17.Ferré, N. and Olivucci, M. Probing the rhodopsin cavity with reduced retinal models at the CASPT2//CASSCF/AMBER level of theory. *J Am Chem Soc* **125,** 6868-9 (2003).doi:10.1021/ja035087d

18.Ferre, N., Cembran, A., Garavelli, M. and Olivucci, M. Complete-active-space self-consistent-field/Amber parameterization of the Lys296--retinal--Glu113 rhodopsin chromophore-counterion system. *Theoretical Chemistry Accounts* **112,** 335-341 (2004).

19.Senn, H. M. and Thiel, W. QM/MM methods for biomolecular systems. *Angew Chem Int Ed Engl* **48,** 1198-229 (2009).doi:10.1002/anie.200802019

20.Ferré, N. and Ángyán, J. G. Approximate electrostatic interaction operator for QM/MM calculations. *Chemical physics letters* **356,** 331-339 (2002).

21.Melaccio, F., Olivucci, M., Lindh, R. and Ferré, N. Unique QM/MM potential energy surface exploration using microiterations. *International Journal of Quantum Chemistry* **111,** 3339-3346 (2011).

22.Aquilante, F., De Vico, L., Ferré, N., Ghigo, G., Malmqvist, P. A., Neogrády, P., Pedersen, T. B., Pitonák, M., Reiher, M., Roos, B. O., Serrano-Andrés, L., Urban, M., Veryazov, V. and Lindh, R. MOLCAS 7: the next generation. *J Comput Chem* **31,** 224-47 (2010).doi:10.1002/jcc.21318

23.Singh, U. C. and Kollman, P. A. A combined ab initio quantum mechanical and molecular mechanical method for carrying out simulations on complex molecular systems: Applications to the CH3Cl+ Cl- exchange reaction and gas phase protonation of polyethers. *Journal of Computational Chemistry* **7,** 718-730 (1986).

24.Hermans, J., Xia, X., Zhang, L. and Cavanaugh, D. Dowser program. *University of North Carolina* (2014).at <http://danger.med.unc.edu/hermans/dowser/dowser.htm>

25.Dundas, J., Ouyang, Z., Tseng, J., Binkowski, A., Turpaz, Y. and Liang, J. CASTp: computed atlas of surface topography of proteins with structural and topographical mapping of functionally annotated residues. *Nucleic Acids Res* **34,** W116-8 (2006).doi:10.1093/nar/gkl282

26.Warshel, A. and Chu, Z. T. Nature of the Surface Crossing Process in Bacteriorhodopsin: Computer Simulations of the Quantum Dynamics of the Primary Photochemical Event. *J Phys Chem B* **105,** 9857-9871 (2001).doi:10.1021/jp010704aat <http://dx.doi.org/10.1021/jp010704a>

27.Gao, J. Hybrid quantum and molecular mechanical simulations: an alternative avenue to solvent effects in organic chemistry. *Accounts of chemical research* **29,** 298-305 (1996).

28.Andersson, K., Malmqvist, P. A., Roos, B. O., Sadlej, A. J. and Wolinski, K. Second-order perturbation theory with a CASSCF reference function. *Journal of Physical Chemistry* **94,** 5483-5488 (1990).

29.Ghigo, G., Roos, B. O. and Malmqvist, P. K. A modified definition of the zeroth-order Hamiltonian in multiconfigurational perturbation theory (CASPT2). *Chemical physics letters* **396,** 142-149 (2004).

30.Gozem, S., Schapiro, I., Ferré, N. and Olivucci, M. The molecular mechanism of thermal noise in rod photoreceptors. *Science* **337,** 1225-8 (2012).doi:10.1126/science.1220461

31.Dartnall, H. J. A. and Lythgoe, J. N. The spectral clustering of visual pigments. *Vision research* **5,** 81-100 (1965).

32.Besalú, E. and Bofill, J. M. On the automatic restricted-step rational-function-optimization method. *Theoretical Chemistry Accounts* **100,** 265-274 (1998).

33.Bowmaker, J. K., Govardovskii, V. I., Shukolyukov, S. A., Zueva, L. V., Hunt, D. M., Sideleva, V. G. and Smirnova, O. G. Visual pigments and the photic environment: the cottoid fish of Lake Baikal. *Vision Res* **34,** 591-605 (1994).

34.Coto, P. B., Strambi, A., Ferré, N. and Olivucci, M. The color of rhodopsins at the ab initio multiconfigurational perturbation theory resolution. *Proc Natl Acad Sci U S A* **103,** 17154-9 (2006).doi:10.1073/pnas.0604048103

35.Altun, A., Yokoyama, S. and Morokuma, K. Mechanism of spectral tuning going from retinal in vacuo to bovine rhodopsin and its mutants: multireference ab initio quantum mechanics/molecular mechanics studies. *J Phys Chem B* **112,** 16883-90 (2008).

36.Andruniów, T., Ferré, N. and Olivucci, M. Structure, initial excited-state relaxation, and energy storage of rhodopsin resolved at the multiconfigurational perturbation theory level. *Proc Natl Acad Sci U S A* **101,** 17908-13 (2004).doi:10.1073/pnas.0407997101

37.Rinaldi, S., Melaccio, F., Gozem, S., Fanelli, F. and Olivucci, M. Comparison of the isomerization mechanisms of human melanopsin and invertebrate and vertebrate rhodopsins. *Proc Natl Acad Sci U S A* **111,** 1714-9 (2014).doi:10.1073/pnas.1309508111

38.Ala-Laurila, P., Donner, K. and Koskelainen, A. Thermal activation and photoactivation of visual pigments. *Biophys J* **86,** 3653-62 (2004).doi:10.1529/biophysj.103.035626

39.Luo, D. G., Yue, W. W., Ala-Laurila, P. and Yau, K. W. Activation of visual pigments by light and heat. *Science* **332,** 1307-12 (2011).doi:10.1126/science.1200172

40.Yanagawa, M., Kojima, K., Yamashita, T., Imamoto, Y., Matsuyama, T., Nakanishi, K., Yamano, Y., Wada, A., Sako, Y. and Shichida, Y. Origin of the low thermal isomerization rate of rhodopsin chromophore. *Sci Rep* **5,** 11081 (2015).doi:10.1038/srep11081

41.Baylor, D. A., Matthews, G. and Yau, K. W. Two components of electrical dark noise in toad retinal rod outer segments. *J Physiol* **309,** 591-621 (1980).

42.Guo, Y., Sekharan, S., Liu, J., Batista, V. S., Tully, J. C. and Yan, E. C. Unusual kinetics of thermal decay of dim-light photoreceptors in vertebrate vision. *Proc Natl Acad Sci U S A* **111,** 10438-43 (2014).doi:10.1073/pnas.1410826111

43.Zaman, M. H., Sosnick, T. R. and Berry, R. S. Temperature dependence of reactions with multiple pathways. *Physical Chemistry Chemical Physics* **5,** 2589-2594 (2003).
